# Spectral, Anti-Inflammatory, Anti-Pyretic, Leishmanicidal, and Molecular Docking Studies, Against Selected Protein Targets, of a New Bisbenzylisoquinoline Alkaloid

**DOI:** 10.3389/fchem.2021.711190

**Published:** 2021-12-17

**Authors:** Muhammad Alamzeb, William N. Setzer, Saqib Ali, Behramand Khan, Mamoon-Ur- Rashid, Ihsanullah, Syed Muhammad Salman, Adnan, Muhammad Omer, Javed Ali, Asad Ullah

**Affiliations:** ^1^ Department of Chemistry, University of Kotli, Kotli, Pakistan; ^2^ Department of Chemistry, University of Alabama in Huntsville, Huntsville, AL , United States; ^3^ Department of Chemistry, Islamia College University, Peshawar, Pakistan; ^4^ Department of Chemistry, Baluchistan University of Information Technology, Engineering and Management Sciences (BUITEMS), Takatu Campus, Quetta, Pakistan; ^5^ Institute of Chemical Sciences, University of Swat, Swat, Pakistan; ^6^ Department of Chemistry, Kohat University of Science and Technology (KUST), Kohat, Pakistan

**Keywords:** chondrofolinol, bisbenzylisoquinoline, Berberis glaucocarpa, molecular docking, bioassay studies 3

## Abstract

A new bisbenzylisoquinoline named as chondrofolinol **(1)** and four reported compounds **(2–5)** were isolated and characterized from the roots of *Berberis glaucocarpa* Stapf. Anti-inflammatory, anti-pyretic, and leishmanicidal studies were performed against carrageenan-induced paw edema, yeast-induced pyrexia, and the promastigotes of *Leishmania tropica*, respectively. The new compound significantly reduced the paw volume in carrageenan-induced paw edema and rectal temperature in yeast-induced pyrexia at 10 and 20 mg/ kg of body weight. Chondrofolinol caused almost 100% inhibition of the promastigotes of *Leishmania tropica*. All the compounds displayed minimal cytotoxicity against THP-1 monocytic cells. In order to ascertain the potential macromolecular targets of chondrofolinol responsible for the observed anti-inflammatory and anti-leishmanial activities, a molecular docking study was carried out on relevant protein targets of inflammation and *Leishmania*. Protein targets of human endoplasmic reticulum aminopeptidase 2 (ERAP2) and human matrix metalloproteinase-1 (MMP-1) for inflammation and protein targets of *N*-myristoyltransferase (NMT), tyrosyl-tRNA synthetase (TyrRS), and uridine diphosphate-glucose pyrophosphorylase (UGPase) for *Leishmania major* were selected after thorough literature search about protein targets responsible for inflammation and *Leishmania major.* Chondrofolinol showed excellent docking to ERAP2 and to MMP-1. The *Leishmania major* protein targets with the most favorable docking scores to chondrofolinol were NMT, TyrRS, and UGPase. The study indicated that bisbenzylisoquinoline and isoquinoline alkaloids possess anti-pyretic, anti-inflammatory, and anti-leishmanial properties with minimal cytotoxicity and therefore, need to be further explored for their therapeutic potential.

## Introduction

Bisbenzylisoquinoline alkaloids contain two benzyl isoquinoline units connected directly through carbon–carbon bond or indirectly through ether or methylenoxy linkages. Their structural diversity arises due to variation in the number, position, and nature of ether or carbon–carbon linkages and number of aromatic oxygenated substituents. They have shown anti-leishmanial, anti-protozoal, anti-trypanosomal, anti-malarial, anti-microbial, cardiovascular, immunomodulatory, anti-influenza, anti-inflammatory, anti-cancer, and cytotoxic properties (Schiff, 1991). Pyrexia is body’s natural response to numerous factors including tissue damage, malignancy, cytokinesis, inflammation, and tumor necrosis factor α (TNF-α). These conditions trigger the hypothalamus to elevate body temperature (Gupta et al., 2011). Fever is associated with depression, lethargy, insomnia, inability to concentrate, and anorexia. Anti-pyretics are used for regulating body temperature which requires a delicate balance between body temperature and hypothalamus (Ochi et al., 2009). Disturbances of homeostasis are associated with inflammations, triggered by innate immune receptors in response to damaged cells and pathogens (Ashley et al., 2012).

About 20 *Leishmania* species are pathogenic to humans. The commonly used front-line anti-leishmanial drugs *viz*. sodium stibogluconate, pentamidine, paramomycin, amphotericin-B, miltefosine, and pentavalent antimony (Grogl et al., 2013) possess several pharmacological drawbacks including prolonged usage, painful administration, toxicity, and resistance by leishmanial parasites. These shortcomings necessitate the quest for new potent and safe drugs (Hamill, 2013).

Inflammation is strongly related with histocompatibility complex class I (MHC-I) alleles. Four inflammatory disorders, namely psoriasis, birdshot chorioretinopathy, ankylosing spondylitis, and Behcet’s disease, are directly associated with MHC-I. MHC-I alleles are directly influenced by endoplasmic reticulum aminopeptidase 1 (ERAP1) and endoplasmic reticulum aminopeptidase 2 (ERAP2) (López de Castro et al., 2016). ERAP2 is structurally related to Zn-metallopeptidases consisting of four domains. Its catalytic site is situated in domain II and a cavity of domain IV. Probably, conformational changes in the orientation of amino acids and domain rearrangements govern the enzymatic activity of ERAP2 (López de Castro, 2018). Matrix metalloproteinase-1 (MMP-1) is the protease which causes the degradation of the extracellular matrix. MMP-1 is one of the key enzymes involved in the process of fibrolysis. Increased levels of MMP-1 have been observed in almost all the diseases involving inflammation. MMP-1 regulates both pathological and normal inflammatory processes. Although inflammation is necessary for tissue repair processes and defense of the host, excessive inflammation can result in organ dysfunction, injury to tissues, and chronic diseases (Manicone and McGuire, 2008). *N-*myristoyltransferase (NMT) is one of the most extensively studied drug targets in Kinetoplastida. NMT catalyzes the attachment of myristate, a 14-carbon fatty acid, to the amino-terminal glycine residue of eukaryotic proteins. The literature search has shown that NMT is a potential candidate for drug development against parasitic protozoan infections such as *Leishmania major.* NMT plays a vital role in mediating protein–protein interactions and targeting protein to membrane locations (Brannigan et al., 2010). Aminoacyl tRNA synthetases (aaRSs) are enzymes which perform protein translation, and hence they play a vital role in the survival of an organism. Tyrosyl-tRNA synthetase (TyrRS) is one of such important aaRSs. TyrRS is believed to be an important target for the development of anti-leishmanial drugs (Barros-Álvarez et al., 2017). Uridine diphosphate-glucose pyrophosphorylase (UGPase) catalyzes the reversible reactions through an ordered sequential Bi–Bi reaction mechanism, which is absolutely vital for the synthesis of monosaccharides and polysaccharides. UGPase is mainly found in the cytoplasm and microsomes of animal cells. UGPases are considered essential for the development of effective anti-leishmanial drugs (Persat et al., 1983; Prakash et al., 2019).

Herein, we report isolation of a new bisbenzylisoquinoline alkaloid named as chondrofolinol **(1)** and four reported compounds **(2–5)** from the root bark of *Berberis glaucocarpa* Stapf along with their *in vivo* anti-pyretic and anti-inflammatory and *in vitro* anti-leishmanial properties and molecular docking studies against the abovementioned selected protein targets.

## Results and Discussion

**FIGURE 1:**
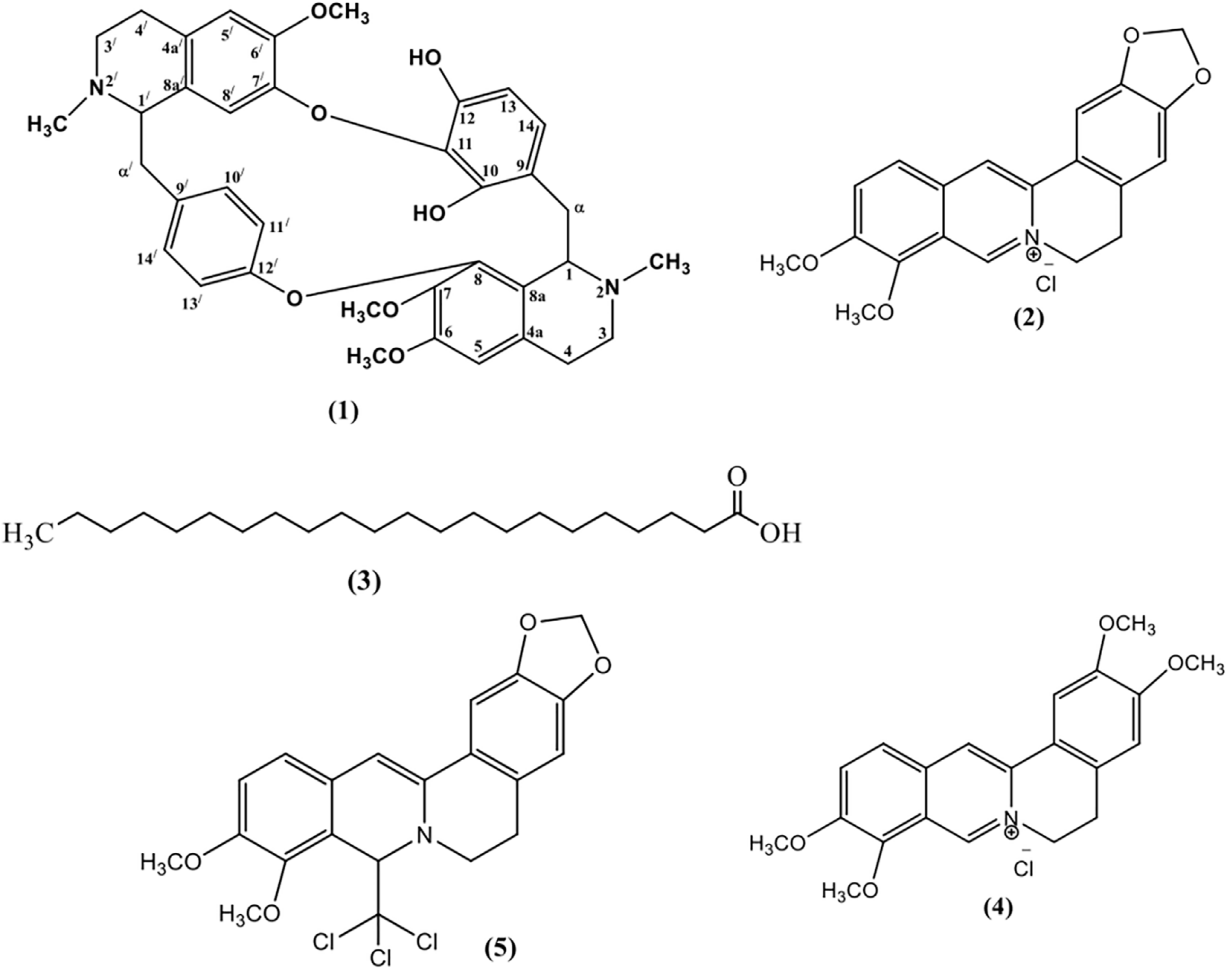
Structures of the isolated compounds 1–5.

One new bisbenzylisoquinoline alkaloid named as chondrofolinol (**1**) and four previously reported compounds **(2–5)**, identified as berberine (**2**) (Grycova et al., 2007), docosanoic acid (**3**) (Hsieh et al., 2004), palmatine (**4**) (Marek et al., 2003), and 8-trichloromethyldihydroberberine (**5**) (Marek et al., 2003), were isolated from the root bark of *Berberis glaucocarpa* (Figure 1). Structures of the isolated compounds were determined from 1D and 2D NMR spectral data.

**FIGURE 2:**
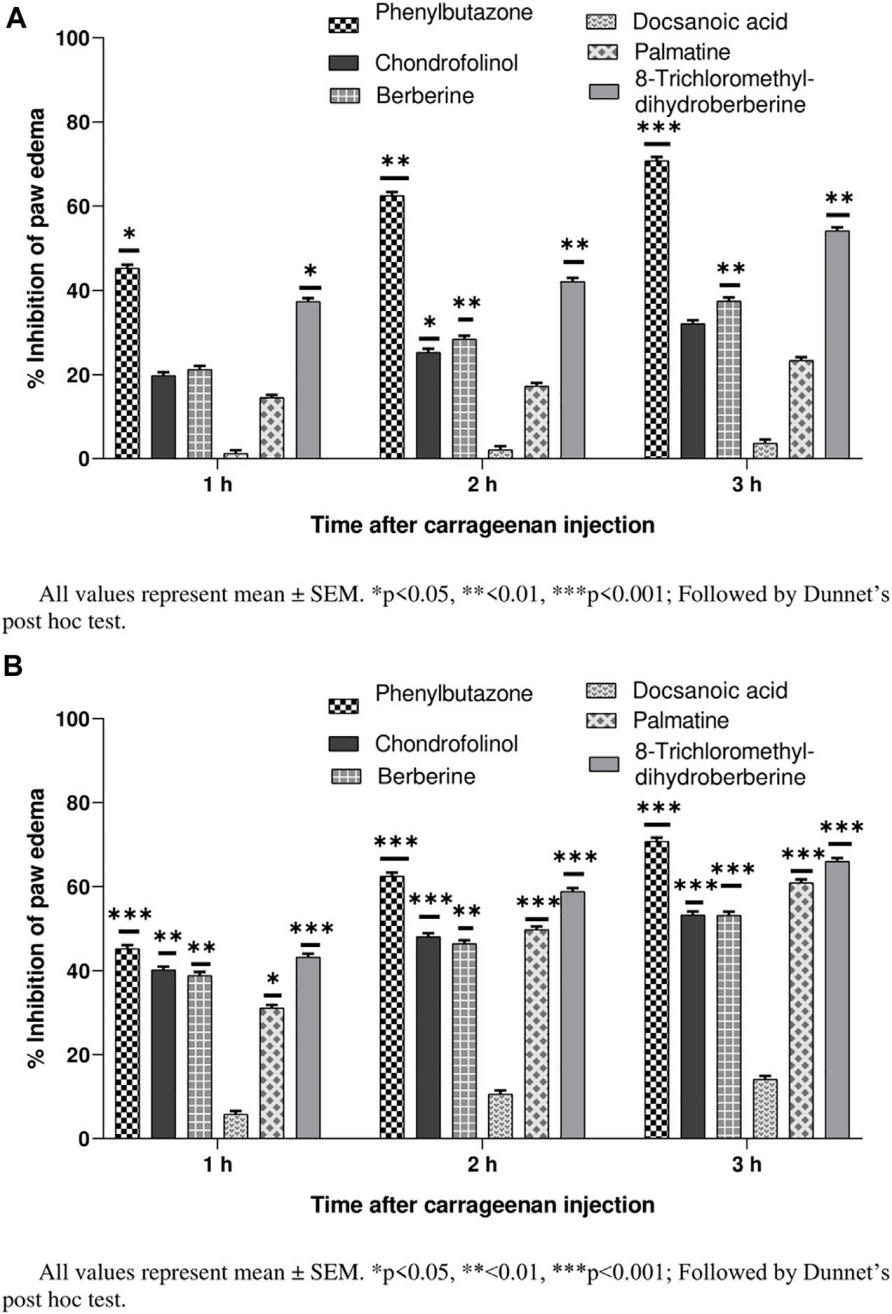
**(A)** Anti-inflammatory potential of compounds 1-5 at 10 mg/ kg. **(B)** Anti-inflammatory potential of compounds 1–5 at 20 mg/ kg.

Their percent inhibitions calculated after 1, 2, and 3 h (24 h after carrageenan injection) indicated mild to moderately strong (32.12–65.91%) anti-inflammatory properties for all compounds except 3 (Figures 2A,B). Chondrofolinol showed significant inhibition (*p* < 0.05) right after the carrageenan injection (32.12% and 53.34% for 10 and 20 mg/ g of chondrofolinol, respectively). Carrageenan-induced edema in biphasic manner. The initial phase (1–1.5 h) was predominantly a non-phagocytic edema, and the second phase (persisted up to 3 h) was an increased edema formation (Khan et al., 2015).

Comprehensive phagocytic inflammation is observed at the 3^rd^ hour, which is followed by carrageenan injection with a large number of neutrophils and tissue edema (Zia-Ul-Haq et al., 2013). Isoquinoline and bisbenzylisoquinoline alkaloids are potent inhibitors of inflammation-producing TNF-α and cytokines. The alkaloids showed marked anti-inflammatory potential possibly by inhibiting the bradykinin peptides. The bio-reduction of bisbenzylisoquinolines to benzylisoquinolines under *in vivo* conditions may be responsible for their anti-inflammatory action (Seow et al., 1993). Hence, the anti-inflammatory potential of chondrofolinol may be attributed to its bio-degradation to benzyl derivatives.

**FIGURE 3:**
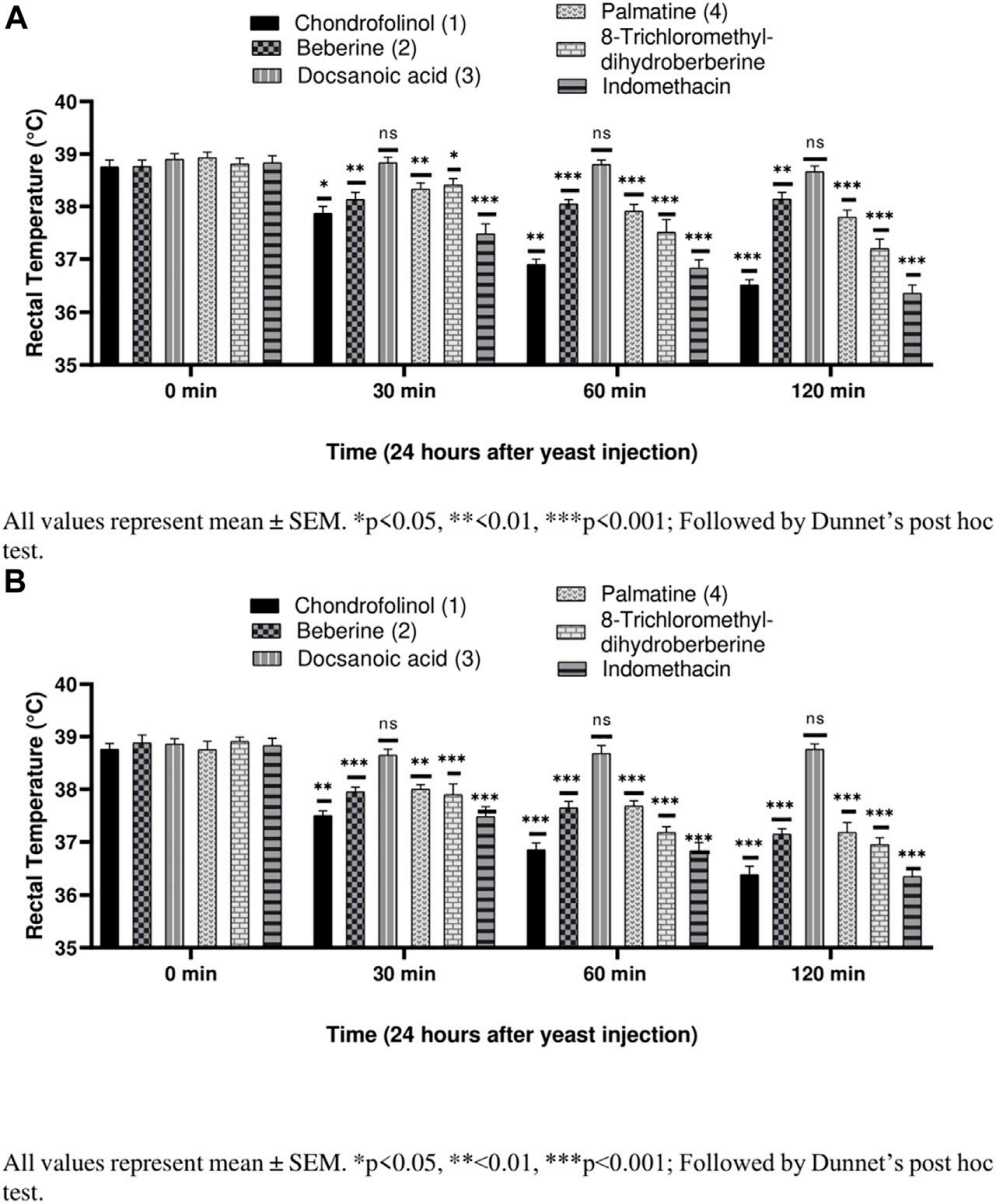
**(A)** Anti-pyretic potential of compounds 1–5 at 10 mg/kg. **(B)** Anti-pyretic potential of compounds 1–5 at 20 mg/kg.

Compounds **1**, **2**, **4**, and **5** were significantly active against yeast-induced hyperthermia (Figures 3A,B). Most of the anti-pyretics exert their effects by inhibiting the cyclooxygenases consequently lowering the levels of PGE_2_ within the hypothalamus. Brewer’s yeast-induced pyrexia, also called pathogenic fever, is due to increased production of PGE_2_ which sets the thermoregulatory center at a higher temperature (Alzubier and Okechukwu, 2011). The anti-pyretic potential of all the isolated compounds (**1**–**5**), against yeast-induced hyperthermia, was calculated after 30, 60, and 120 min after 3 h of brewer’s yeast injection. Chondrofolinol displayed milder anti-pyretic potential at both concentrations (10 and 20 mg/kg).

**FIGURE 4:**
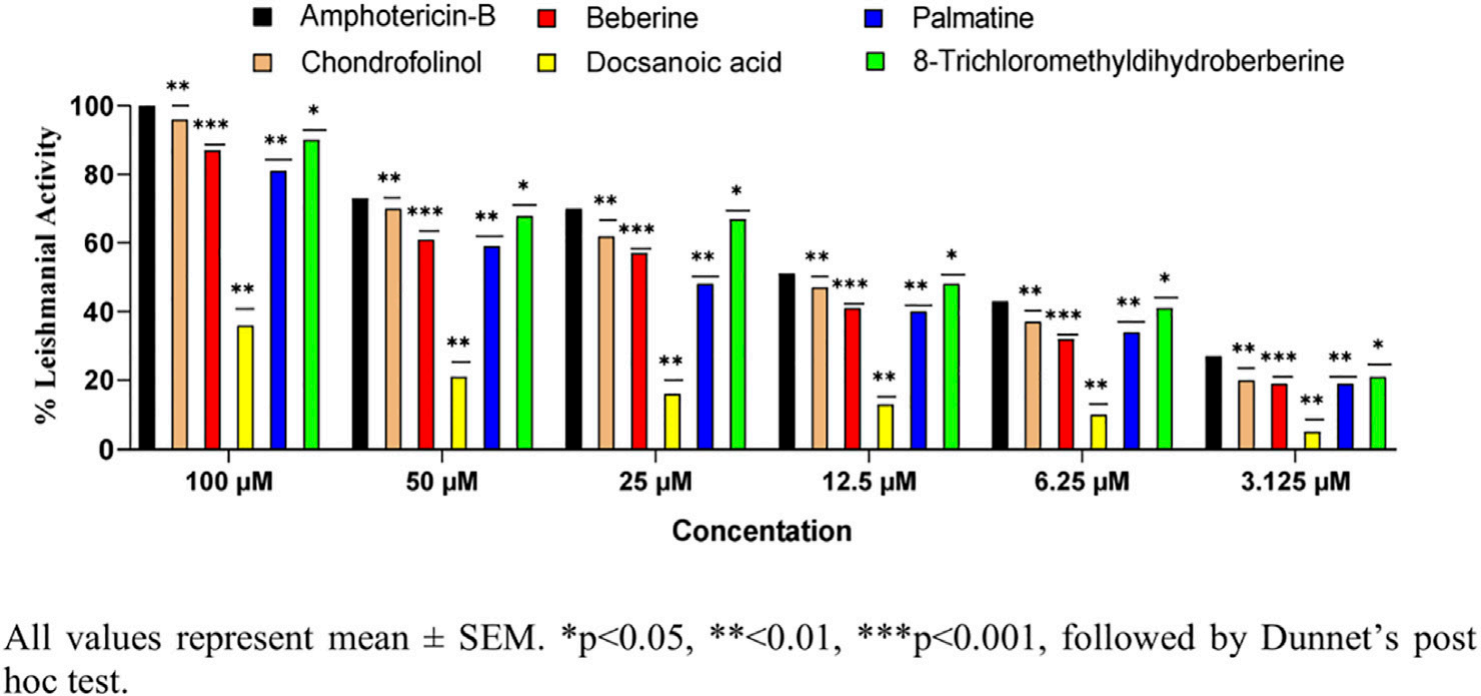
Anti-leishmanial properties of 1–5 against *Leishmania tropica*.

All the isolated compounds were also tested for their anti-leishmanial properties (Figure 4) against clinical field isolates of *Leishmania tropica.* The new isolate **1)** and compounds **2**, **4**, and **5** displayed strong anti-leishmanial properties at all four tested concentrations. These findings are in agreement with anti-leishmanial and anti-inflammatory properties of previously reported bisbenzylisoquinoline alkaloids (Alamzeb et al., 2018). However, their anti-pyretic potentials have seldom been reported.

The docking study was carried out on relevant protein targets of inflammation and *Leishmania* using the Molegro Virtual Docker program (Thomsen and Christensen, 2006). *In silico* screening of chondrofolinol was carried out against molecular targets of inflammation, including ovine COX-1 (Oa COX-1), murine COX-2 (Mm COX-2), murine soluble epoxide hydrolase 2 (Mm EPHX2), human soluble epoxide hydrolase 2 (Hs EPHX2), human endoplasmic reticulum aminopeptidase 2 (Hs ERAP2), human glutathione transferase omega 1 (HsGSTO1), human inhibitor of nuclear factor kappa-B kinase subunit beta (Hs IKKβ), *Xenopus laevis* IKKβ, human interleukin-1 receptor–associated kinase 4 (Hs IRAK4), murine-inducible nitric oxide synthase (Mm iNOS), human c-Jun *N*-terminal kinase (Hs JNK), human 5-lipoxygenase (Hs5-LOX), human myeloid differentiation protein-2 (Hs MD-2), human fibroblast collagenase (matrix metalloproteinase-1, Hs MMP-1), human myeloperoxidase (Hs MPO), murine nuclear factor kappa-light-chain-enhancer of activated B cells (Mm NF-κB), human P38 mitogen-activated protein kinase (Hs p38MAPK), human phosphodiesterase 4B (Hs PDE4B), human phosphodiesterase type 4D (Hs PDE4D), human phosphoinositide 3-kinase gamma (Hs PI3Kγ), human pancreatic secretory phospholipase A2 (Hs PLA2), porcine pancreatic phospholipase A2 (Ss PLA2), and human peroxisome proliferator–activated receptor gamma (Hs PPAR-γ). The docking energies of chondrofolinol with inflammatory target proteins are shown in Supplementary Table S2.

**FIGURE 5:**
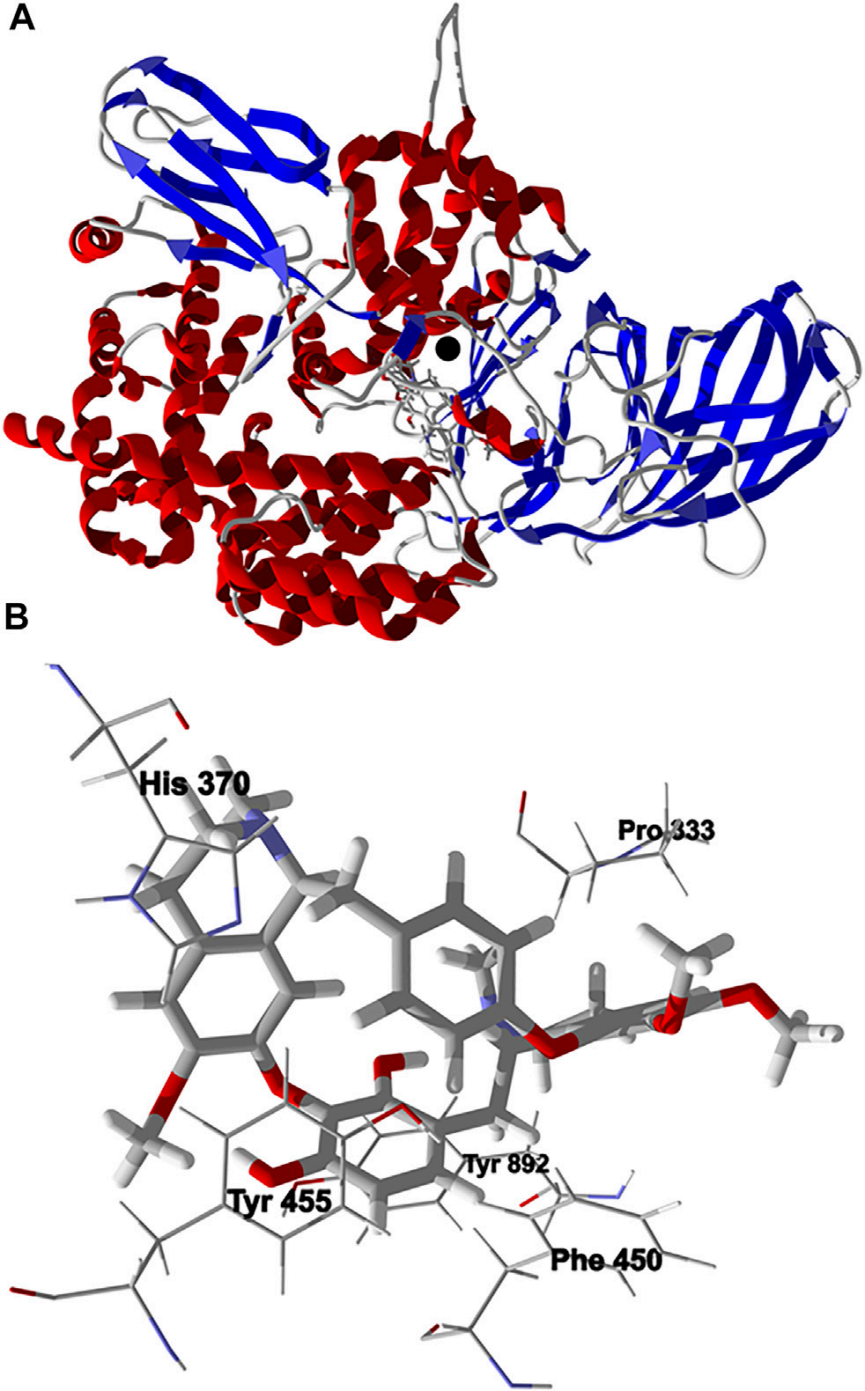
Lowest-energy docked pose of chondrofolinol with human endoplasmic reticulum aminopeptidase 2 (Hs ERAP2, PDB 4JBS). **(A)**: Ribbon structure (the Z^2+^ ion is shown as a black sphere and the docked ligand is shown as a stick figure). **(B)**: Binding site showing key intermolecular interactions of chondrofolinol with the protein.

Chondrofolinol showed relatively favorable docking to human endoplasmic reticulum aminopeptidase 2 (Hs ERAP2, *E*_dock_ = −132.7 kJ/ mol) and to human matrix metalloproteinase-1 (Hs MMP-1, *E*_dock_ = −119.3 kJ/ mol). The binding site of ERAP2, a Zn^2+^ metalloprotease, include hydrophobic pockets surrounded by Tyr892, Tyr455, and Phe450, and an active site occupied by Zn^2+^ complexed by His370 and His374 (Zervoudi et al., 2013). The lowest-energy docked pose of chondrofolinol with ERAP2 (PDB 4JBS) shows key interactions with Pro333, Tyr892, Tyr455, His370, and Phe450 (Figure 5). The docked chondrofolinol is also near to the Zn^2+^ cofactor; closest interatomic distance 3.42 Å.

**FIGURE 6:**
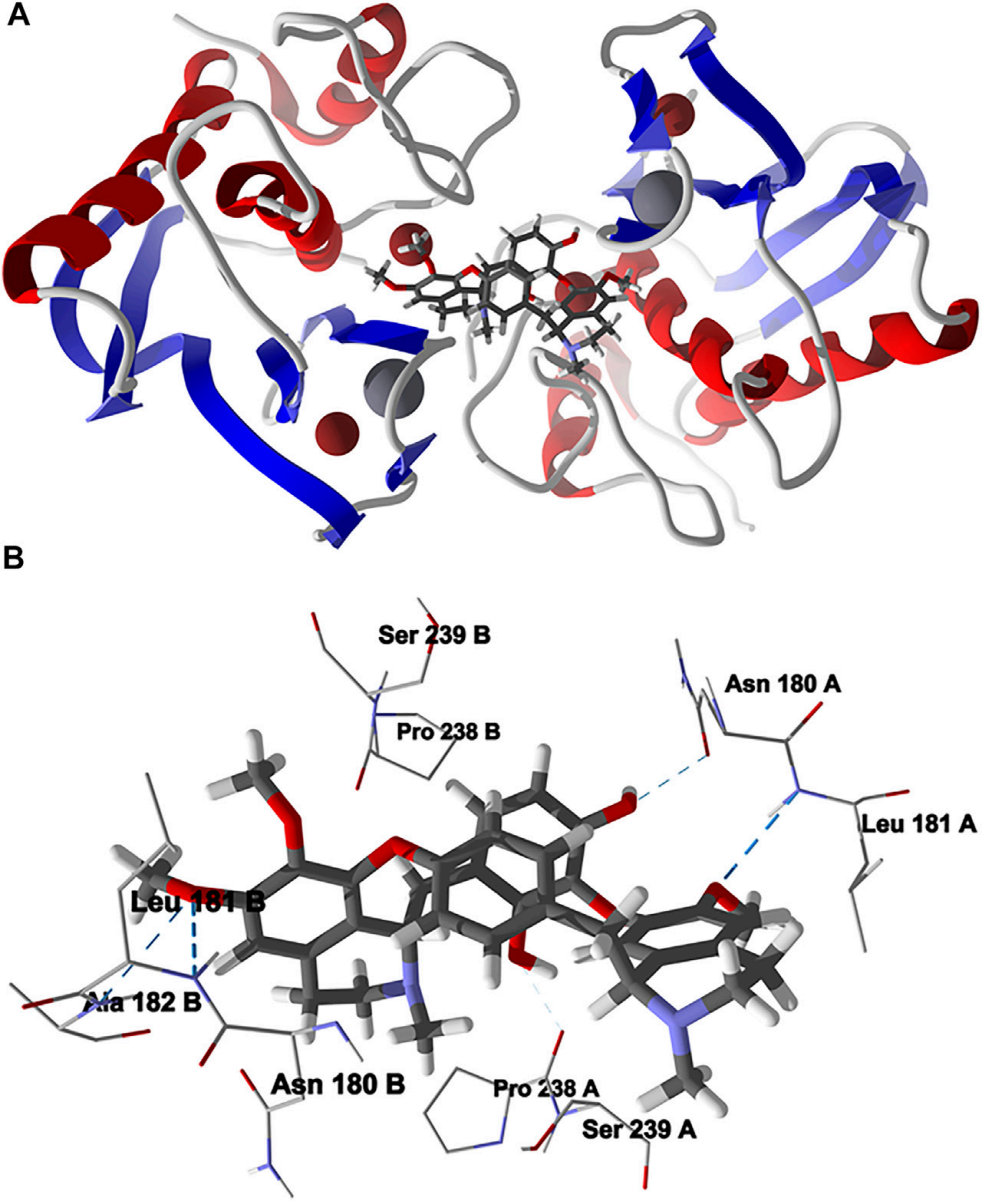
Lowest-energy docked pose of chondrofolinol with human matrix metalloproteinase-1 (Hs MMP-1, PDB 1CGL). **(A)**: Ribbon structure (Zn^2+^ ions are shown as dark red spheres, Ca^2+^ ions are shown as gray spheres, and the docked chondrofolinol is shown as a stick figure). **(B)**: Active site showing the interactions of important contacts in the active site with the docked ligand (hydrogen-bonded interactions are indicated with blue dashed lines).

The lowest-energy docked pose of chondrofolinol with human matrix metalloproteinase-1 (Hs MMP-1, PDB 1CGL) is shown in Figure 6. The ligand occupies the interface between the two monomers of the dimeric protein structure. The key interactions of the ligand with the protein are with Asn180B, Pro238 A (H-bonding), Ser239A, Leu181B (H-bonding), Pro238B, Leu181 A (H-bonding), Asn 180 A (H-bonding), Ser239B, and Ala182B (H-bonding).

The favorable docking scores of chondrofolinol with ERAP2 and with MMP-1 suggest that these may be anti-inflammatory targets of chondrofolinol. Molecular docking of chondrofolinol was carried out with several *Leishmania* protein targets (Ogungbe et al., 2013) (Supplementary Table S3.). The *Leishmania* protein targets with the most favorable docking scores to chondrofolinol were Lmaj NMT (*E*_dock_ = −122.0 kJ/ mol), Lmaj TyrRS (*E*_dock_ = −115.9 kJ/ mol), Lmaj UGPase (*E*_dock_ = −114.9 kJ/ mol), Lmaj OPB (*E*_dock_ = -107.9 kJ/ mol), and Lmaj PYK (*E*_dock_ = −107.8 kJ/ mol).

**FIGURE 7:**
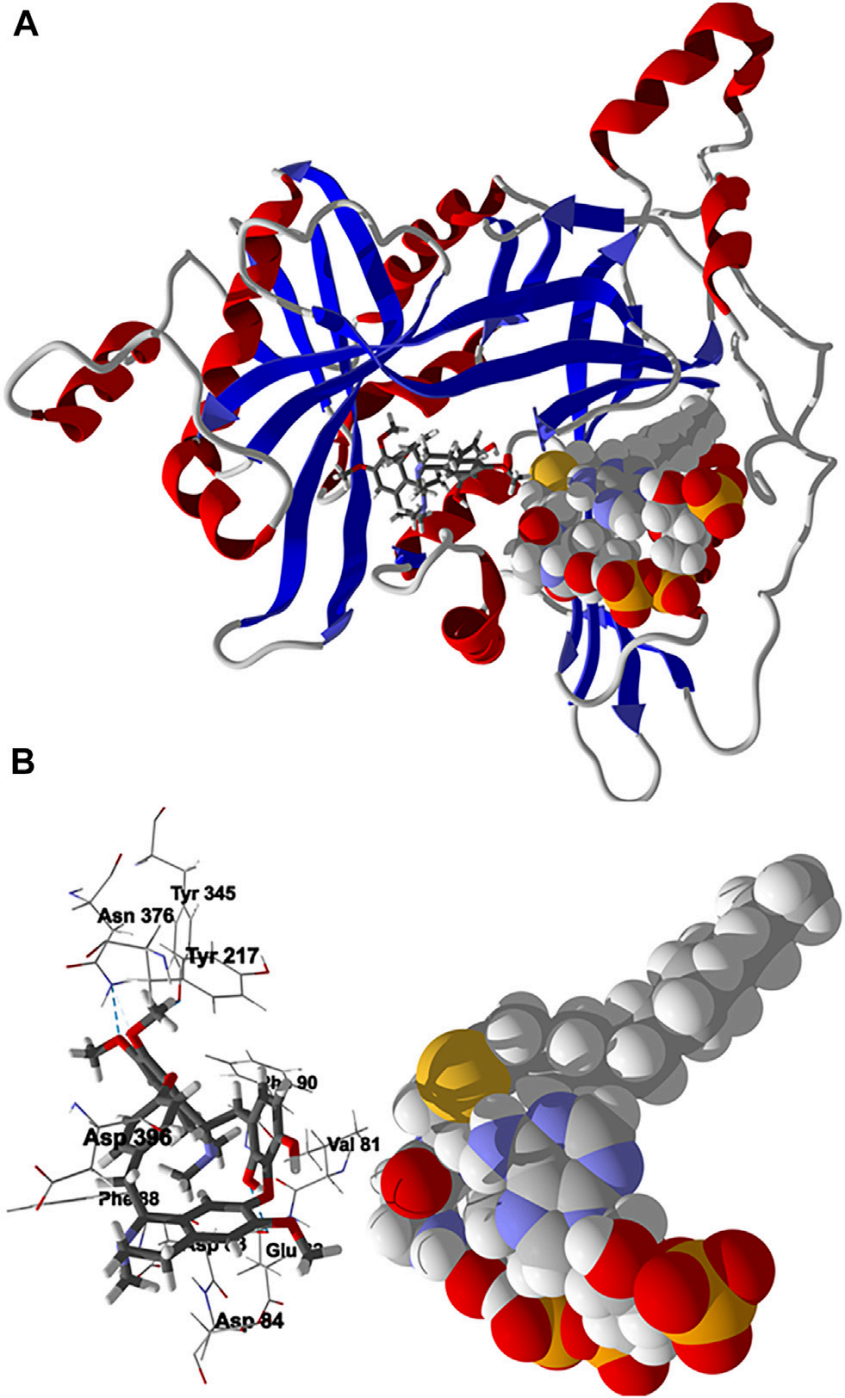
Lowest-energy docked pose of chondrofolinol with *Leishmania major N*-myristoyltransferase (Lmaj NMT, PDB 2WSA). **(A)**: Ribbon structure (the myristoyl CoA cofactor is shown as a space-filling structure and the docked chondrofolinol is shown as a stick figure). **(B)**: Active site showing the interactions of important contacts in the active site with the docked ligand (hydrogen-bonded interactions are indicated with blue dashed lines).

**FIGURE 8:**
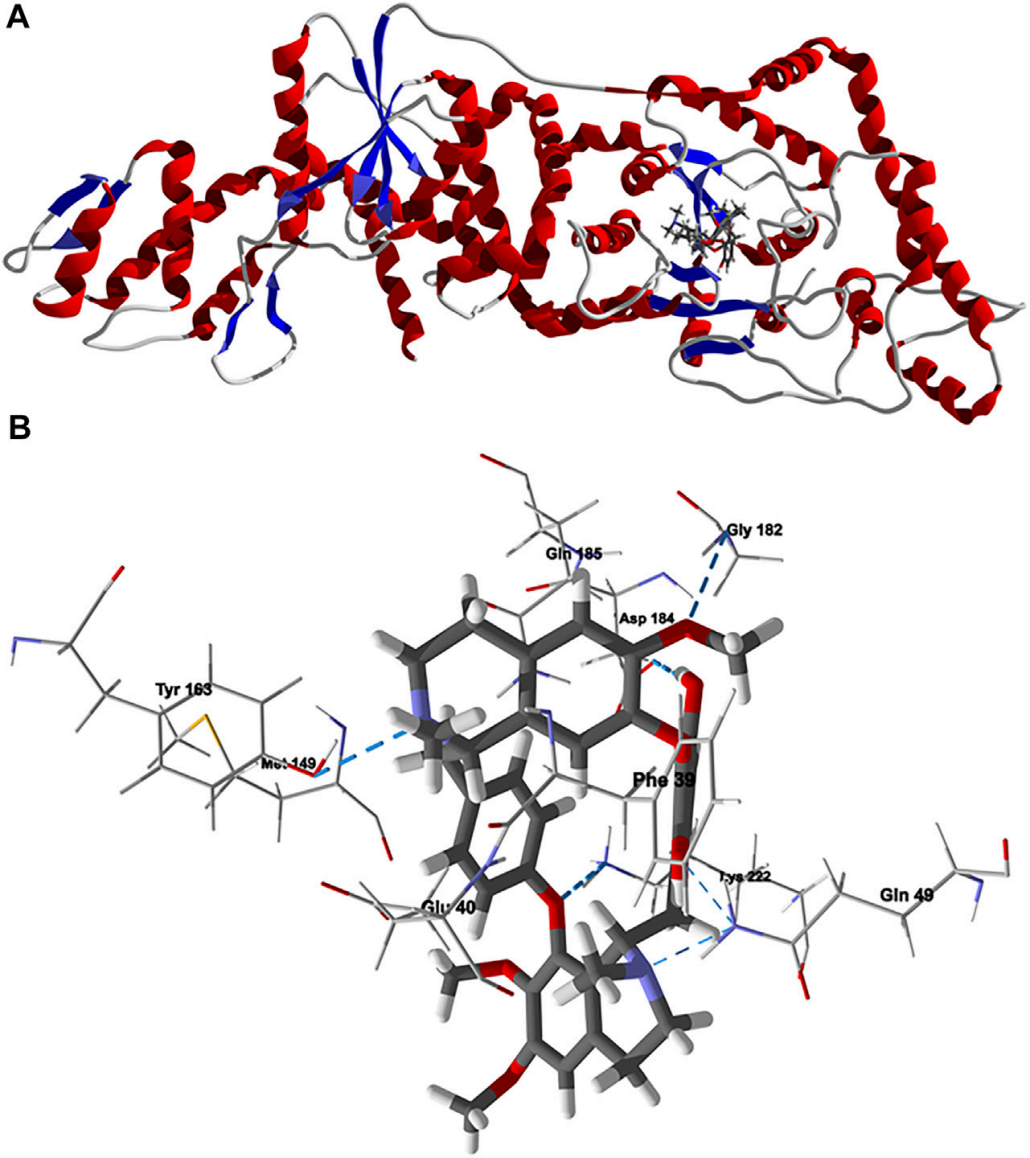
Lowest-energy docked pose of chondrofolinol with *Leishmania major* tyrosyl-tRNA synthetase (Lmaj TyrRS, PDB 3P0J). **(A)**: Ribbon structure (the docked chondrofolinol is shown as a stick figure). **(B)**: Active site showing the interactions of important contacts in the active site with the docked ligand (hydrogen-bonded interactions are indicated with blue dashed lines).

**FIGURE 9:**
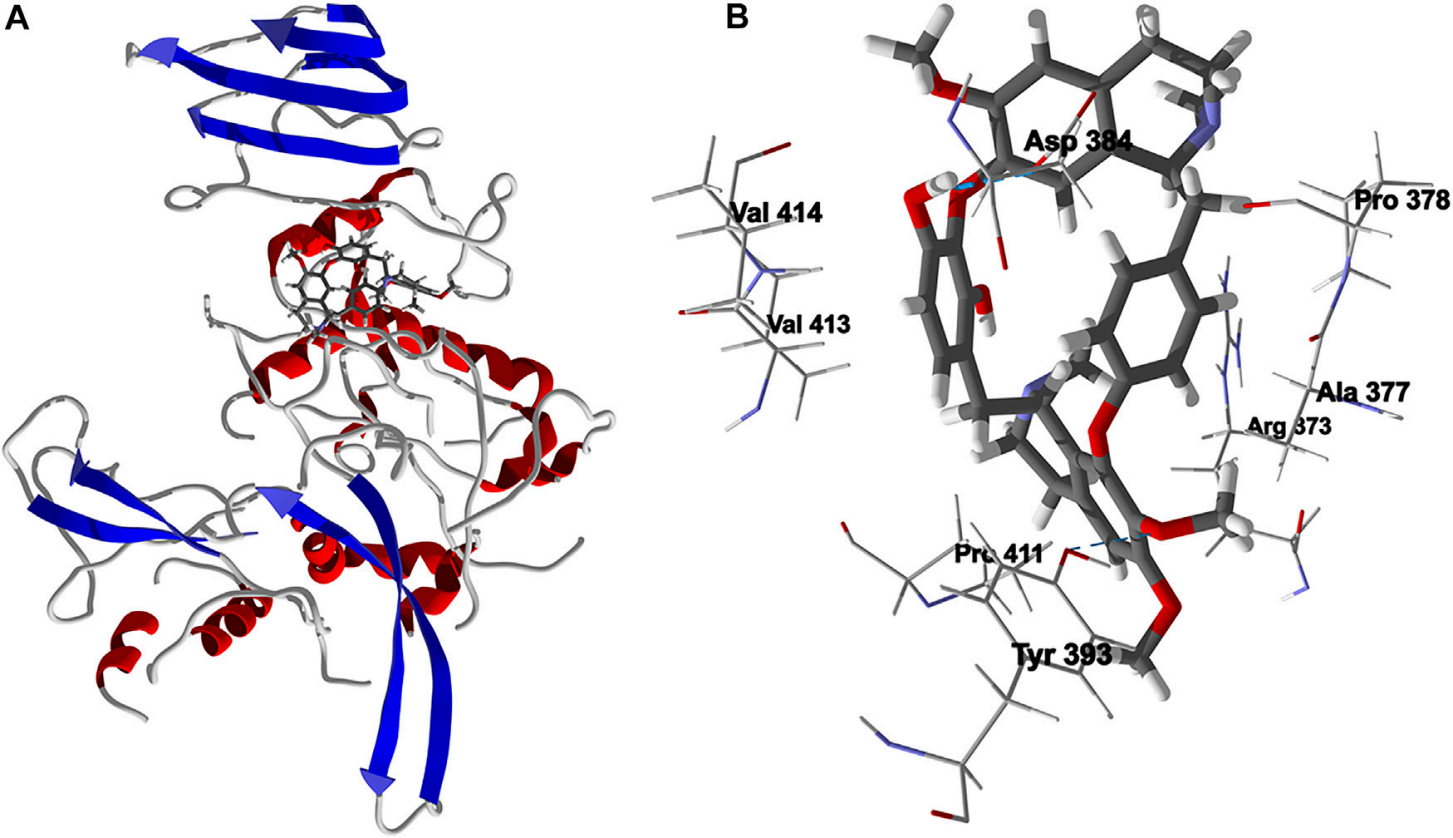
Lowest-energy docked pose of chondrofolinol with *Leishmania major* uridine diphosphate-glucose pyrophosphorylase (Lmaj UGPase, PDB 2OEF). **(A)**: Ribbon structure (the docked chondrofolinol is shown as a stick figure). **(B)**: Active site showing the interactions of important contacts in the active site with the docked ligand (hydrogen-bonded interactions are indicated with blue dashed lines).

**FIGURE 10:**
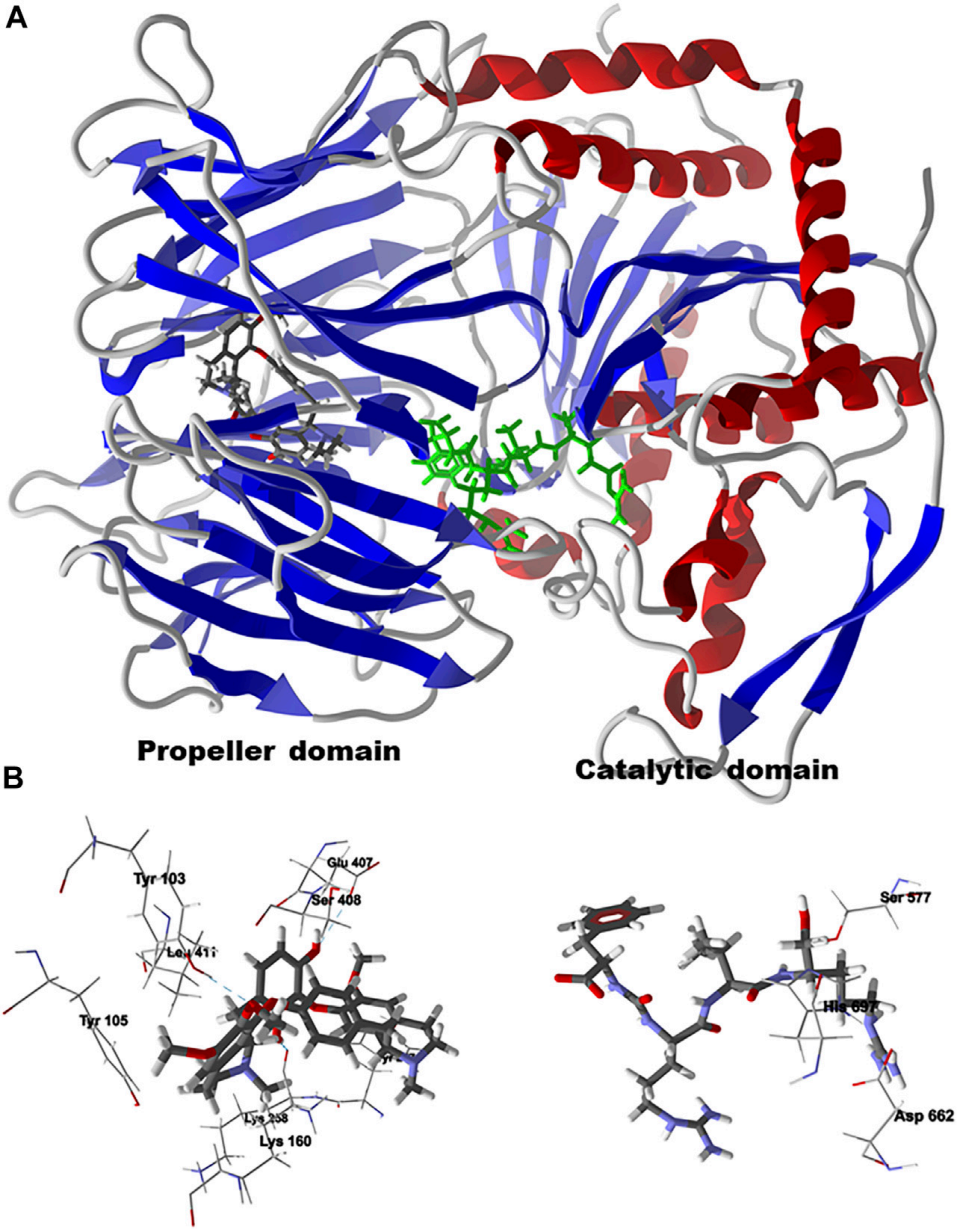
Lowest-energy docked pose of chondrofolinol with *Leishmania major* oligopeptidase B (Lmaj OPB, PDB 2XE4). **(A)**: Ribbon structure (the docked chondrofolinol is shown as a CPK stick figure and the co-crystallized antipain is shown as a green stick figure). **(B)**: Preferred docking site of chondrofolinol showing the key intermolecular contacts (hydrogen-bonded interactions are indicated with blue dashed lines); the co-crystallized ligand, antipain, is also shown in the active site along with the catalytic triad.

Chondrofolinol docked preferentially in the active site of the enzyme in proximity to the myristoyl CoA co-factor (Figure 7A). The key intermolecular contacts between Lmaj NMT and chondrofolinol are Phe90, Asp84, Asp396, Asp83, Glu82 (H-bonding), Tyr217, Val81, Phe88, Asn376 (H-bonding), and Tyr345 (H-bonding) (Figure 7B). *Leishmania* tyrosyl-tRNA synthetase (TyrRS), one of the aminoacyl-tRNA synthetases (Ibba and Soll, 2000), is an essential enzyme in protein translation. The lowest-energy pose of chondrofolinol occupies the active site of the enzyme with key interactions with amino acid residues Gln185, Lys222 (H-bond), Glu40, Gln49 (H-bond), Met149, Phe39, Tyr163 (H-bond), Asp184 (H-bond), and Gly182 (H-bond) (Figure 8). The active site of *L. major* UGPase was occupied by chondrofolinol with a docking score of -114.9 kJ/ mol. The main intermolecular interactions between the protein and the ligand were Asp384 (H-bonding), Arg373, Pro411, Pro378, Val413, Val414, Ala377, and Tyr393 (H-bonding) (Figure 9). *Leishmania* oligopeptidase B (OPB) is a serine protease, which is a virulence factor in *Trypanosoma brucei* and *T. cruzi*. However, the lowest-energy docked pose for chondrofolinol is removed from the active site of the enzyme and occupies the so-called propeller domain of the protein (McLuskey et al., 2010). Figure 10 shows the docked site of chondrofolinol in *L. major* OPB (PDB 2XE4) as well as the co-crystallized ligand antipain in the active site. If chondrofolinol is an inhibitor of *L. major* OPB, it is likely an allosteric effector.

**FIGURE 11:**
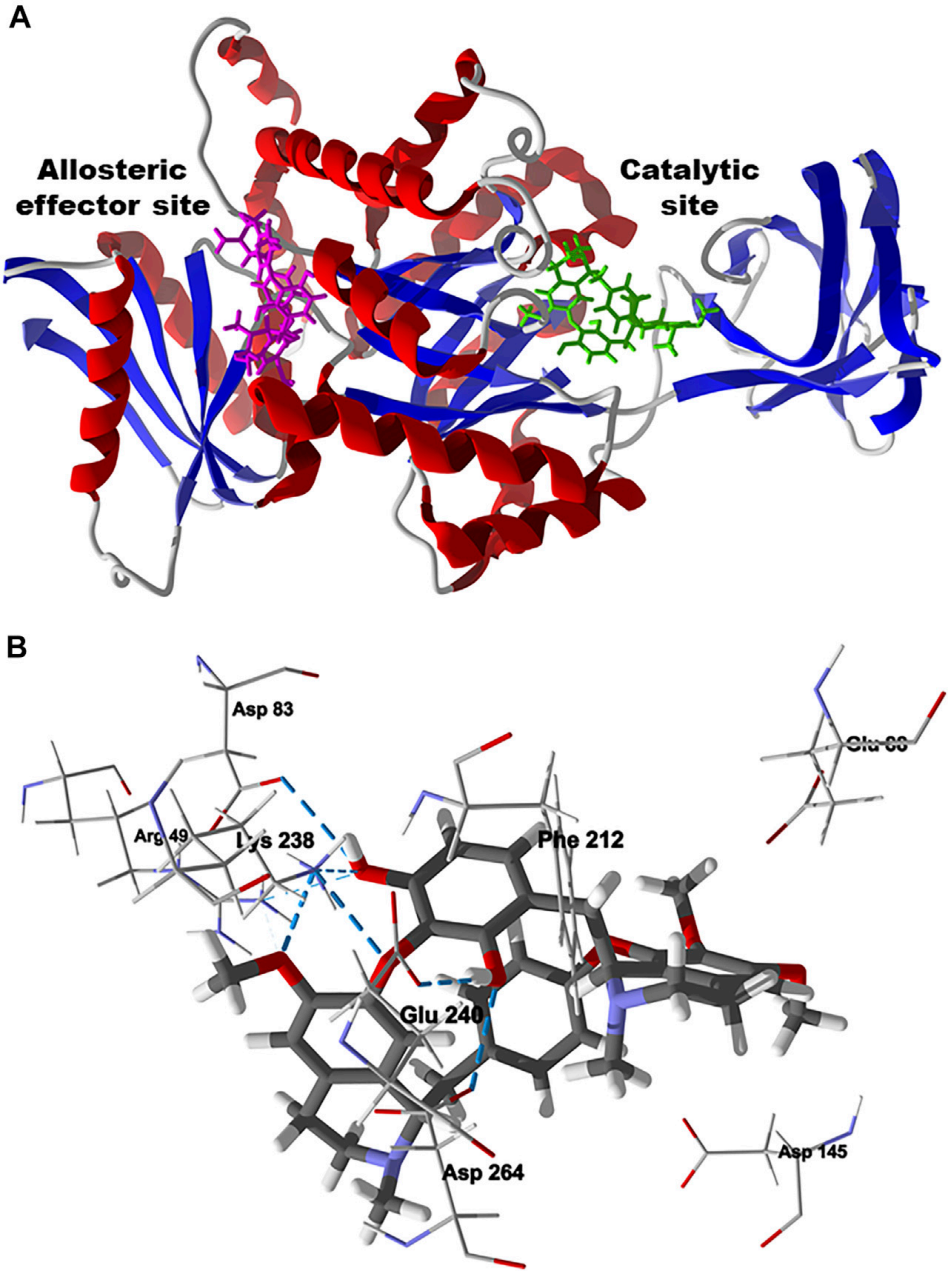
Lowest-energy docked poses of chondrofolinol with *Leishmania mexicana* pyruvate kinase (Lmex PYK, PDB 1PKL). **(A)**: Ribbon structure showing the docked chondrofolinol poses in the allosteric effector site (magenta stick figure) and the catalytic site (green stick figure). **(B)**: Lowest-energy docked pose of chondrofolinol showing the key intermolecular contacts (hydrogen-bonded interactions are indicated with blue dashed lines).

The preferred docking site of chondrofolinol with *L. mexicana* PYK is the active site (*E*_dock_ = −107.8 kJ/ mol) rather than the allosteric effector site (*E*_dock_ = -86.8 kJ/mol) (Figure 11A) (Rigden et al., 1999). The important contacts between chondrofolinol and the active site of *L. mexicana* PYK are Asp264, Asp145, Phe212, Glu88, Lys238 (H-bonding), Glu240 (H-bonding), Arg49 (H-bonding), and Asp83 (H-bonding) (Figure 11B). Although chondrofolinol has shown very favorable docking scores with several *Leishmania* protein targets, the anti-leishmanial activity may also be due to interactions with additional *Leishmania* proteins or other biochemical targets that are yet to be elucidated.

## Experimental

### Chemistry Procedures

Merck Kieselgel silica gel 60 PF_254_ (70–230 mesh ASTM) and Sephadex LH-20 were used for column chromatography (CC) and thin-layer chromatography (TLC). Melting points were determined on the Stuart digital melting point apparatus (SMP 10) and were uncorrected. Ultraviolet/visible spectra were recorded on the Thermo Spectronic Unicam UV-300. Infrared spectra were recorded on JASCO FTIR- 4200 A and Thermo Scientific FTIR Nicolet-380. Electron impact ionization mass spectrometry (EIMS) was carried out with the JEOL MS-Route instrument. High-resolution electrospray ionization mass spectrometry (HR-ESIMS) was performed on the Thermo Scientific Exactive LCQ fleet instrument. ^1^HNMR, ^13^CNMR, and 2D NMR were recorded on Bruker Avance DRX-400 and 500 MHz and JEOL 400 MHz instruments.

### Plant Material

*Berberis glaucocarpa* Stapf roots were collected from Azad Kashmir, Pakistan (34° 23*′* 16.0044“ N and 73° 32*′* 51.9900"*”* E.), during the flowering period, and after identification voucher specimen number 9615-A was deposited in the herbarium of Botany Department University of Peshawar.

### Extraction and Isolation

The root bark (2 kg) was first dried at room temperature, pulverized, and then extracted with commercial grade methanol. This, upon concentration with a rotary evaporator, yielded a dark brownish black residue (172 g). The residue was then treated with 5% aqueous HCl solution to afford fraction **A**. The filtrate was then extracted with dichloromethane to afford fraction **B** (9 g). The acidic filtrate was then basified with NH_3_ to pH 9 and extracted with ethyl acetate to afford fraction **C** (31 g). After repeated column and preparative chromatography of fractions **B** and **C,** one new bisbenzylisoquinoline alkaloid (**1**) and four reported compounds (**2–5**) were isolated and characterized from the root barks of *Berberis glaucocarpa*.

### Structure Determination and Elucidation of Chondrofolinol

Chondrofolinol (**1**) was obtained as an off-white amorphous powder. The molecular formula C_37_H_39_N_2_O_7_ was deduced from the HR-ESIMS (Supplementary Figure S1) peak at *m/z* 623.2772 [M-H]^+^ (calculated for C_37_H_39_N_2_O_7_, 623.2772), implying 19 degrees of unsaturation. The UV/Vis spectrum (λ_max_ (log ε)): 282 nm (4.27) indicated the presence of benzylisoquinoline moieties (Supplementary Figure S2). Its IR spectrum (Supplementary Figure S3) suggested the presence of hydroxy group (3,400–3,300 cm^−1^) and aromatic ring (1,595 and 1,500 cm^−1^) functionalities.

The ^1^HNMR spectrum (Supplementary Table S1, Supplementary Figure S4) of **1** in the lower field showed the presence of a para-disubstituted benzene ring at *δ*_H_ 7.29 (IH, dd, *J* = 8.4, 2.4 Hz, H-10^**/**^), 7.13 (1H, dd, *J* = 8.4, 2.4 Hz, H-11^**/**^), 6.43 (1H, dd, *J* = 8.4, 2.4 Hz, H-14^**/**^), and 6.64 (1H, dd, *J* = 8.4, 2.4 Hz, H-13^**/**^); an unusual 1,2,3,4-tetrasubstituted benzene ring at *δ*_H_ 6.79 (1H, d, *J* = 8.0 Hz, H-13) and 6.75 (1H, d, *J* = 8.0 Hz, H-14); and three aromatic singlet protons at *δ*_H_ 6.53 (1H, s, H-5^**/**^), 6.28 (1H, s, H-5), and 5.99 (1H, br. s*,* H- 8^**/**^). Five heteroatom-bearing singlet methyls [*δ*_H_ 3.76 (s, 6-OMe); 3.59 (s, 6^**/**^-OMe); 3.12 (3H, s, 7-OMe), 2.58 (s, 2-*N*-Me)_;_ and 2.26 (s, 2^/^-*N*-Me)] were observed in the higher field. The DEPTq-135 spectrum (Supplementary Table S1, Supplementary Figure S5) revealed the presence of thirty-seven carbon signals, including the signals corresponding to the aforementioned units, six methylenes (*δ*_C_ 45.69, C-3^**/**^; 38.58, C-α; 25.34, C-4; 45.01, C-3; 37.66, C-α^**/**^ **;**24.97, C-4^**/**^), two methines (δ_C_ 63.68, C-1; 62.23, C-1^**/**^), two hydroxyl-attached carbons (δ_C_ 147.63, C-10; 148.20, C-12), and 15 aromatic quaternary carbons.

The HMBC correlations (Supplementary Table S1, Supplementary Figure S6) from H-4 to C-3, C-5, C-8a, and C-4a, from H-1 to C- α, N-Me, C-4a, and C-8, from H-5 to C-4a, C-8a, C-6, C-7, and C-4, and from 2-*N*-CH_3_ (*δ*_H_ 2.26) to C-1 and C-3 were used to construct ring-A and ring-B. Similarly, the HMBC correlations from H-5^**/**^to C-4^**/**^a, C-6^**/**^, C-7^**/**^, C-8^**/**^a, and C-4^**/**^, from H-8^**/**^to C-1^**/**^, C-4^**/**^a, C-7^**/**^ **,**and C-6^**/**^, and from H-1^**/**^to C- α^/^, N^/^-Me, C-3^/^, C-8^/^, C-8^/^a, C- 4^/^a, and C-9^/^ constructed the ring-A^**/**^and ring-B^**/**^. HMBC and NOESY spectra were used for establishing the positions of the three methoxy groups. The COSY (Supplementary Figure S7) and NOESY (Supplementary Figure S8) spectra of compound **1** established (RR) configurations for carbons 1 and 1^**/**^. Comparison of the UV λ_max_ value and ^1^HNMR with the literature (Guha et al., 1979; Cometa et al., 2012; Wang et al., 2020) affirmed chondrofolinol as a head-to-tail bisbenzylisoquinoline alkaloid containing two diphenyl ether bridges C-8/C-12^**/**^and C-11/C-7^**/**^.

### Animals

Albino rats (180–200 g) of either sex were used in all experiments. The animals were kept under standard laboratory conditions (25 °C and light/dark cycles, i.e., 12/12 h) and were fed with standard food and water *ad libitum*.

### Anti-Pyretic Studies

Albino rats of either sex weighing 180–200 g were used for anti-pyretic studies. The animals were divided into five groups with each group having six albino rats (*n* = 6). All the animals were given free access to food and water. Basal rectal temperature was measured using a digital clinical thermometer before injecting the yeast. Hyperthermia was induced in rats by sub-cutaneous (s.c.) injection of 20% aqueous suspension of brewer’s yeast (20 ml/ kg) in the back below the nape of the neck (Loux et al., 1972). Group 1 was treated with normal saline as a control, and group 2 was treated with indomethacin (10 mg/kg, s. c) as the standard drug, while the remaining groups 3–5 were treated with compound **1**–**5** (10 mg/ kg and 20 mg/ kg, s. c). After 24 h from the yeast injection, the rise in the rectal temperature was recorded at regular intervals of 30, 60, and 120 min after drug administration.

### Carrageenan-Induced Paw Edema Studies

Albino rats of either sex (180–200 g) were used to assess anti-inflammatory activity. The animals were randomly divided into five groups with each group having six albino rats (*n* = 6). Pedal inflammations in albino rats of either sex were produced according to the method described by Winter *et al.* (1962). Group 1 was treated with normal saline (5 ml/ kg) and group 2 was treated with oxyphenylbutazone (10 mg/ kg, i.p.), while groups 3–5 were treated with compounds **1**–**5** (10 mg/ kg and 20 mg/ kg, i.p.). After 30 min, 0.05 ml 1% carrageenan sodium salt (BDH) was subcutaneously injected into the plantar aponeurosis tissue of the right hind paw of each rat. The inflammation was measured using a plethysmometer (Apelex, France) immediately after +3 h of carrageenan injection. The anti-inflammatory activity was calculated using the following formula:

$$100\left(1-\frac{a-x}{b-y}\right),$$

where “b” is the mean paw volume of control rats after carrageenan injection and “y” before the injection; whereas “x” is the mean paw volume of treated rats before injection and “a” is the mean paw volume after carrageenan injection.

### Parasite Culture for Leishmanial Studies

The initial culture was raised in the 5 ml M-199 medium supplemented with 10% heat-inactivated fetal bovine serum (HI-FBS), 100 μg/ ml penicillin, 100 μg/ ml streptomycin, 50 μg/ ml kanamycin, and 5 μg/ ml of hemin in a 15-ml culture flask. The culture flask was kept in an incubator at 26°C, and the medium was changed every third day. The cultures were given enough time (10–14 days) to fully develop and promastigotes to reach the metacyclic stage. The culture was then sub-passaged by pouring the contents of the flask containing the promastigotes into a centrifuge tube. The tubes were then spun in a Sigma centrifuge at 2000 rpm for 12 min. After spinning, the medium was carefully poured off leaving the pellet only. This pellet was re-suspended in the 2–4 ml growth medium, and new cultures were established. The primary culture was used for sensitivity assays, and additional cultures were cryopreserved in liquid nitrogen at −196°C for future use.

### Anti-Leishmanial Assays

The culture of *L. tropica* clinical field isolate KWH_23_ was established from already cryopreserved collection in the Department of Zoology, University of Peshawar. The initial culture was raised in the 5 ml M-199 medium supplemented with 10% heat-inactivated fetal bovine serum (HI-FBS), 100 μg/ ml penicillin, 100 μg/ ml streptomycin, 50 μg/ ml kanamycin, and 5 μg/ ml of hemin in a 15-ml culture flask. Two experiments were run at a time under the same conditions in 96-well flat-bottom plates. The promastigotes were counted in culture suspension by using the improved Neubauer hemocytometer. From the viable promastigotes bulk culture (4 × 10^6^/ ml), 1 × 10^5^ promastigotes/well in the 200 µL fresh M-199 medium were seeded in 96-well plates. Four different concentrations for each compound were applied, and one row of 12 wells was kept as a control with the growth medium only. The 96-well plates were incubated at 26°C for 48 h. After 48 h, the number of promastigotes in each well was counted microscopically using the improved Neubauer hemocytometer.

### Culture of THP-1 Cells

THP-1 cells were initially cultured in the complete RPMI 1640 medium. The cells were grown in a carbon dioxide incubator in humidified air with 5% CO_2_ at 37°C. The cells were added with the fresh medium on every third day, and after bulking up, they were harvested for checking the cytotoxicity of the compounds **1-5**.

### Cytotoxicity determination of Compounds 1–5

**FIGURE 12:**
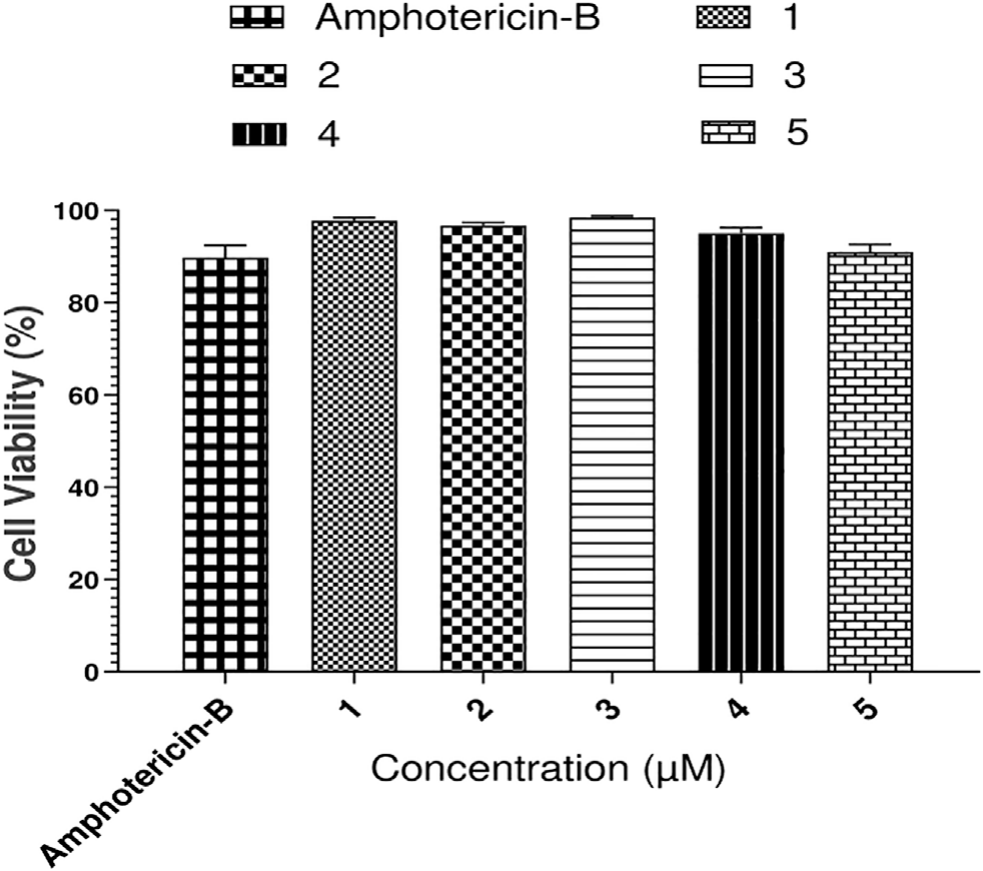
Cytotoxicity results of compounds **1–5** against THP-1 monocytic cells.

For the determination of cytotoxicity of the tested compounds (Figure 12), THP-1 monocytic leukemia cells were used. The THP-1 cells were originally obtained from the London school of Hygiene and Tropical Medicine. The THP-1 cells were maintained in the RPMI 1640 medium (containing 10% HI-FCS, 2 mM l-glutamine, and 1% penicillin and streptomycin) with 5% CO_2_ at 37°C. The THP-1 cells with 100 percent viability were centrifuged at 1,200 rpm, and the supernatant was removed. The fresh medium was added to the cells, and 50,000 cells/200 µL medium/well were added to flat-bottom 96-well plates. For optimization, each compound and the standard (amphotericin-B) were applied in triplicate with six different concentrations (100, 50, 25, 12.50, 6.25, and 3.125 µM). The plate was then placed in a humidified CO_2_ incubator at 37°C. Viability of cells was determined by using a trypan blue exclusion technique after 48 h via improved Neubauer hemocytometer. The viability of cells was determined by the trypan blue exclusion analysis before and after treatment with the drugs. An equal volume of the 0.4% trypan blue reagent was added to the cell suspension, and the percentage of viable cells was evaluated under a dark-field microscope. The assay is based on the principle that viable cells are non-permeable for the dye, while the dead cells lost their membrane property and turned blue. Viability was calculated using the following formula:

$$\%\;\mathrm{viability}=\left(\frac{\mathrm{live}\;\mathrm{cell}\;\mathrm{count}}{\mathrm{total}\;\mathrm{cell}\;\mathrm{count}}\right)\times 100.$$

From these data, percent cell inhibition was determined, and their average was taken.

### Statistical Analysis

The results obtained are expressed as average ±SEM of six animals. The statistical analyses were performed by ANOVA followed by Dunnett’s test for multiple comparisons. *p* < 0.05 was considered significant in the experiments.

### Molecular Docking

The structure of chondrofolinol was prepared using Spartan ’18 for Windows v 1.4.4. The lowest-energy conformation was determined using the Merck molecular force field (MMFF). (Halgren, 1996). Molecular docking was carried out using Molegro Virtual Docker v. 6.0.1 (Molegro ApS, Aarhus, Denmark) (Thomsen and Christensen, 2006) as previously reported (Snow Setzer et al., 2016). A 15-Å sphere was centered on the binding sites of each protein structure in order to permit the ligand to search. Standard protonation states of each protein, based on neutral pH, were used, and charges were assigned based on standard templates as part of the Molegro Virtual Docker program. Each protein was used as a rigid model without protein relaxation. A flexible-ligand model was used in the docking optimizations. The scoring function used is described in Thomsen and Christensen (2006). Different orientations of the ligand were searched and ranked based on their “rerank” docking scores. A total of 100 runs for the ligand with each protein structure were carried out. . In order to test the docking accuracy and to compare docking scores, the co-crystallized ligands were re-docked with the protein structures (see Supplementary Tables S4, S5).

## Conclusion

It can be concluded from this study that the isolated compounds possess anti-pyretic, anti-inflammatory, and anti-leishmanial properties. Molecular docking explains their anti-inflammatory and leishmanicidal potential in terms of interactions between the active sites of enzymes, responsible for inflammation and Leishmania, and isolated alkaloids.

## Data Availability

The original contributions presented in the study are included in the article/Supplementary Material, further inquiries can be directed to the corresponding author.
