# Supplementary material for: Spectral, Anti-Inflammatory, Anti-Pyretic, Leishmanicidal, and Molecular Docking Studies, Against Selected Protein Targets, of a New Bisbenzylisoquinoline Alkaloid
Source: Front Chem. 2021 Dec 17;9:711190. doi: 10.3389/fchem.2021.711190 (PMC8719521; doi:10.3389/fchem.2021.711190)
Supplement: Supplementary file 1 [file Table1.doc]

**Table captions**

**Table S1.** ^1^H NMR (400 MHz, CDCl_3_) DEPTq-135 (100 MHz, CDCl_3_), ^1^H and HMBC (400 MHz, CDCl_3_) data of **Chondrofolinol (1)**

**Table S2.** MolDock docking energies (*E*_dock_, kJ/mol) of chondrofolinol with inflammation-

relevant protein targets.

**Table S3.** MolDock docking energies (*E*_dock_, kJ/mol) of chondrofolinol with *Leishmania* protein

targets.

**Table S4.** MolDock docking energies (*E*_dock_, kJ/mol) of co-crystallized ligands and root-mean-squared deviations (Å) between the co-crystallized ligand and the re-docked poses of the co-crystallized ligand with inflammation-relevant protein targets.

**Table S5.** MolDock docking energies (*E*_dock_, kJ/mol) of co-crystallized ligands and root-mean-squared deviations (Å) between the co-crystallized ligand and the re-docked poses of the co-crystallized ligand with *Leishmania* protein targets.

**Table S1.**

**C. No *δ* _C_ *δ* _H_ (mult, *J* Hz) HMBC (H→C) COSY NOESY**

1 63.68 3.86, (br. s) C- α, N-Me, C-4a, C-8, H- α

C-8a, C-9, C-3

N-Me 41.97 2.26, (s) C-1, C-3

3 45.01 2.87 and 3.46, (m) C-1, N-Me, C-4, C-4a H-4

4 25.34 2.81 and 2.95, (m) C-3, C-5, C-8a, C-4a, H-3

4a 128.71 --- ---

5 105.43 6.28, (s) C-4a, C-8a, C-6, C-7, C-4 H-4, 6-OMe

6 151.77 --- ---

7 136.90 --- ---

6-OMe 55.55 3.76 (s) C-6 H-5

7-OMe 60.51 3.12 (s) C-7

8 148.66 --- ---

8a 120.24 --- ---

α 38.58 2.54-2.51 (dd, 14.8, 10.4) C-1, C-14, C-9, C-8a, C-10 H-1 N-Me

and 3.04-2.97 (dd,14.0, 15.6)

9 133.63 --- ---

10 147.63 --- ---

11 143.44 --- ---

12 148.20 --- ---

13 115.34 6.79, (d, 8.0) C-14, C-9, C-11, C-12 H-14 H- α

14 123.50 6.75, (d, 8.0) C-13, C-9, C-10, C-12, C- α H-13 H- α

1**/** 62.23 3.89, (br. s) C- α/, N/-Me, C-3/, C-8/, H- α

C-8/a, C- 4/a, C-9/

N**/**-Me 42.71 2.58, (s) C-1**/**, C-3**/** H-10**/**

3**/** 45.69 2.83 and 3.27, (m) C-4**/**, N**/**-Me, C-1**/**, C-4**/**a H-4**/**

4**/** 24.97 2.77 and 2.93, (m) C-3**/**, C-1**/**, C-4**/**a, C-5**/** H-3/

4**/**a 127.33 --- ---

5**/** 111.19 6.53, (s) C-4**/**a, C-6**/**, C-7**/**, C-8**/**a, C-4**/** H-3**/**, H-4**/**, 6**/**-OMe

6**/** 149.92 --- ---

6**/**-OMe 55.76 3.59, (s) C-6**/** H**-**5**/**

7**/** 143.89 --- ---

8**/** 119.84 5.99, (br.s) C-1**/**, C-4**/**a, C-7**/**, C-6**/**

8**/**a 127.75 --- ---

α**/** 37.66 2.93-2.87 (dd, 8.0, 9.2) C-1**/**, C-9**/**, C-14**/**,C-10**/** H-1**/**

and 3.34-3.25 (dd, 14.0, 13.2)

9**/** 135.42 --- ---

10**/** 130.22 7.29 (dd 8.4, 2.4) C-11, C-14**/,** C-12**/**, C- α**/** H-11**/** H-11**/**, H- α**/**, H-1/

11**/** 121.35 7.13, (dd, 8.4, 2.4) C-13**/**, C-10**/**, C-9**/**, C-12**/** H-10/ H-10/

12**/** 153.90 --- ---

13/ 121.72 6.64, (dd, 8.4, 2.4) C-11**/**, C-14**/**, C-9**/**, C-12**/** H-14**/** H-14**/**

14**/** 132.71 6.43, (dd, 8.4, 2.4) C-13**/**, C-10**/**, C-12**/** H-13/ H-13/, H- α**/**, H-1/

br.s = broad singlet, dd = doub
