# Supplementary material for: Spectral, Anti-Inflammatory, Anti-Pyretic, Leishmanicidal, and Molecular Docking Studies, Against Selected Protein Targets, of a New Bisbenzylisoquinoline Alkaloid
Source: Front Chem. 2021 Dec 17;9:711190. doi: 10.3389/fchem.2021.711190 (PMC8719521; doi:10.3389/fchem.2021.711190)
Supplement: Supplementary file 2 [file DataSheet1.PDF]

SUPPLEMENTARY MATRIAL

Comprehensive molecular docking against human protein targets to explain and validate the anti-inflammatory, anti-pyretic and leishmanicidal properties of a new bisbenzylisoquinoline alkaloid.

Muhammad Alamzeb[1*], William N. Setzer[2], Saqib Ali[1], Behramand Khan[3], Mamoon-Ur-Rashid[4], Ihsanullah[5], Syed Muhammad Salman[3], Adnan[5], Muhammad Omer[5].

[1] *Department of Chemistry, University of Kotli, Kotli-11100, Azad Jammu and Kashmir, Pakistan.*

[2] *Department of Chemistry, University of Alabama in Huntsville, Huntsville, Alabama 35899, United States.*

[3] *Department of Chemistry, Islamia College University, Peshawar-KPK 25120, Pakistan.*

[4] *Department of Chemistry, Baluchistan University of Information Technology, Engineering and Management Sciences (BUITEMS), Takatu Campus, Quetta-87100, Pakistan.*

[5] *Institute of Chemical Sciences, University of Swat, Swat-19201, Pakistan.*

<u>Table of contents</u>

Figure S1. HR-ESIMS of **Chondrofolinol**

Figure S2. UV spectrum of **Chondrofolinol.**

Figure S3. IR spectrum of Chondrofolinol

Figure S4. 1HNMR spectrum of **Chondrofolinol** in $CDCl_3$

Figure S5. DEPTq 135 spectrum of **Chondrofolinol** in $CDCl_3$

Figure S6. HMBC spectrum of Chondrofolinol in $CDCl_3$

Figure S7. COSY spectrum of Chondrofolinol in $CDCl_3$

Figure S8. NOESY spectrum of Chondrofolinol in $CDCl_3$

Figure S9. HSQC spectrum of Chondrofolinol in $CDCl_3$

Figure S1

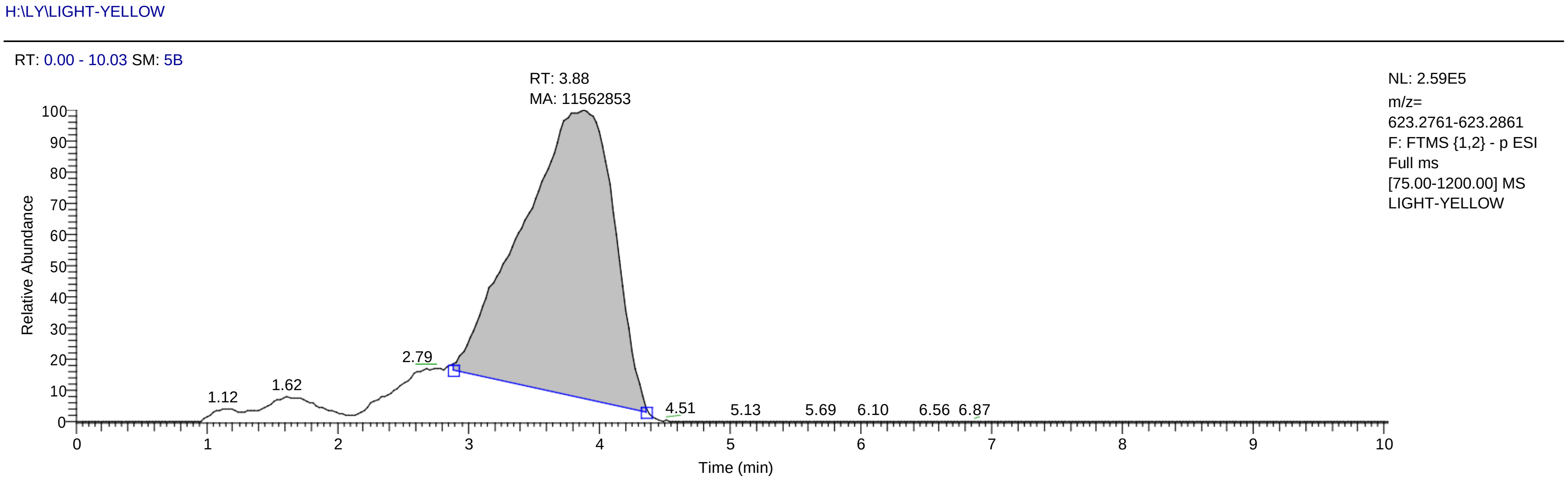
H:\LY\LIGHT-YELLOW
RT: 0.00 - 10.03 SM: 5B
RT: 3.88
MA: 11562853
NL: 2.59E5
m/z=
623.2761-623.2861
F: FTMS {1,2} - p ESI
Full ms
[75.00-1200.00] MS
LIGHT-YELLOW
Relative Abundance
Time (min)
1.12
1.62
2.79
4.51
5.13
5.69
6.10
6.56
6.87

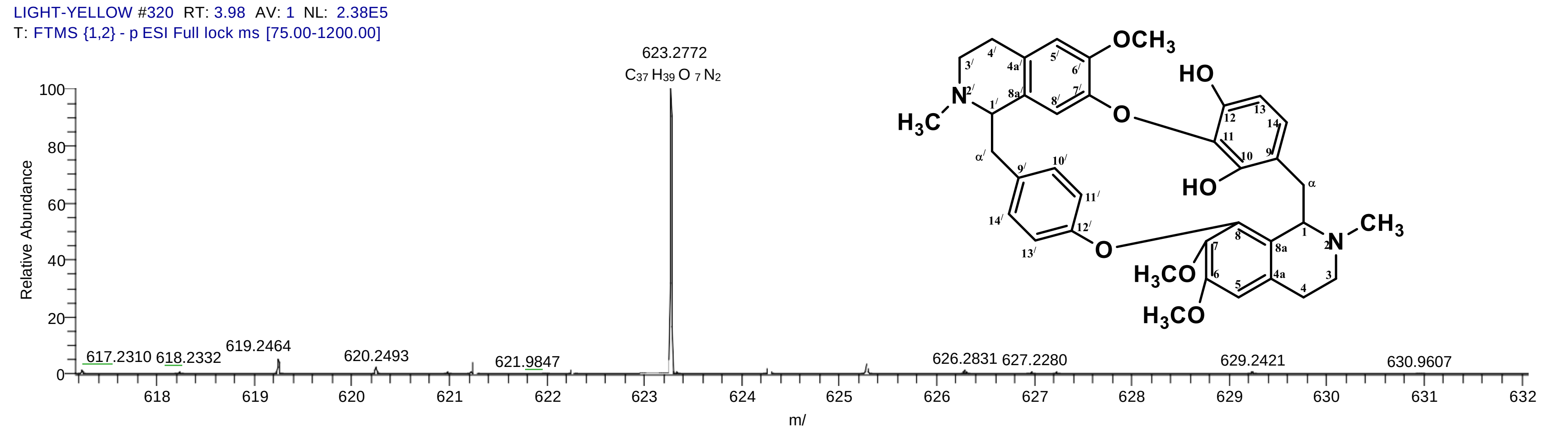
LIGHT-YELLOW #320 RT: 3.98 AV: 1 NL: 2.38E5
T: FTMS {1,2} - p ESI Full lock ms [75.00-1200.00]
623.2772
C37 H39 O 7 N2
Relative Abundance
m/
617.2310
618.2332
619.2464
620.2493
621.9847
626.2831
627.2280
629.2421
630.9607
OCH3
HO
HO
H3C
CH3
H3CO
H3CO
N
N
O
O

Figure S 2

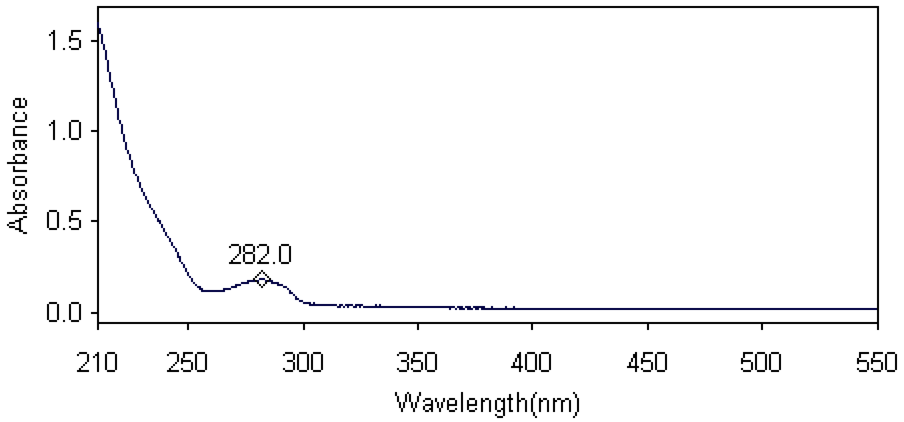
282.0
1.5
1.0
0.5
0.0
Absorbance
210
250
300
350
400
450
500
550
Wavelength(nm)

Figure S 3

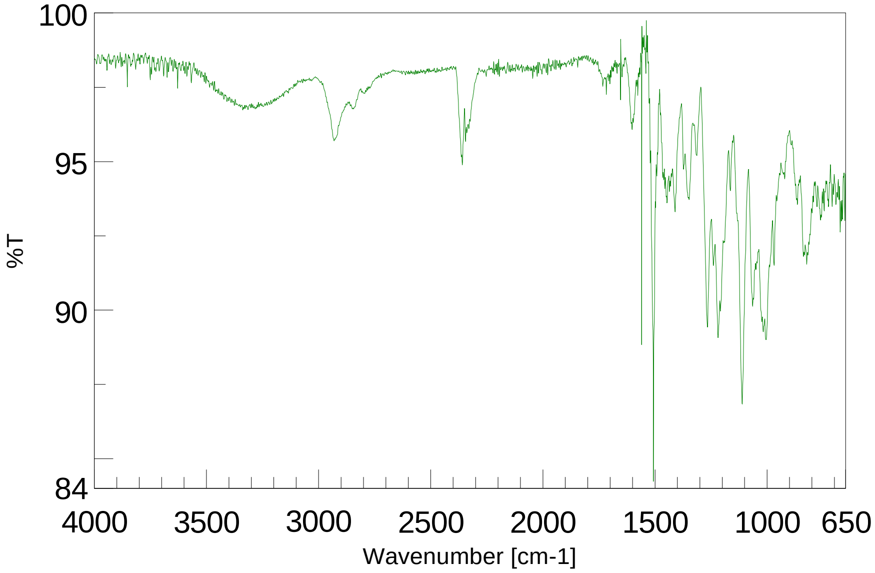

100
95
90
84
%T
4000
3500
3000
2500
2000
1500
1000
650
Wavenumber [cm-1]

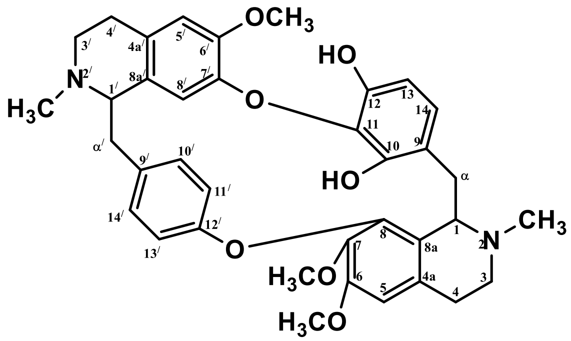

Figure S 4

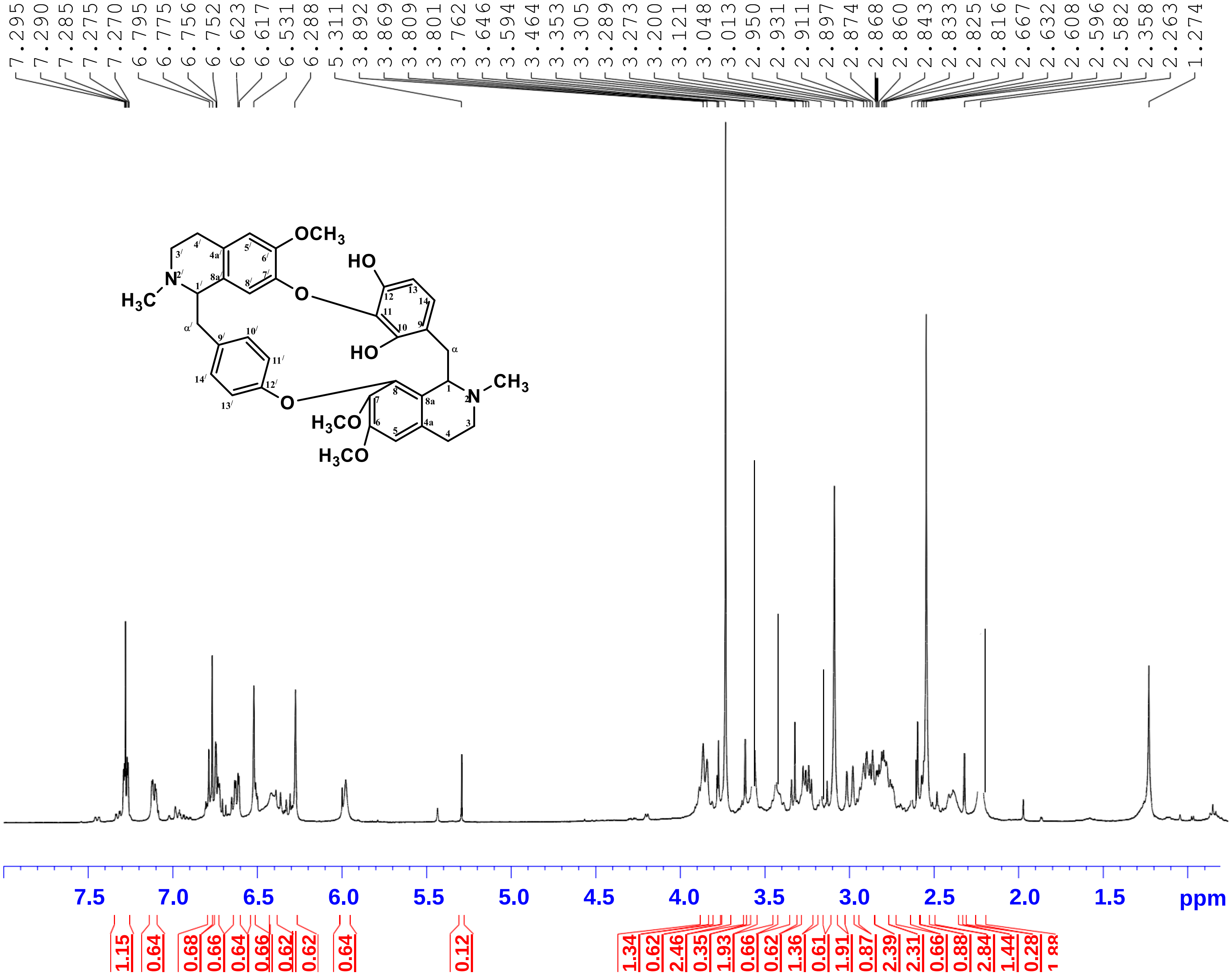

NAME	D162831
EXPNO	1
PROCNO	1
Date_	20120608
Time	13.17
INSTRUM	spect
PROBHD	5 mm QNP 1H/13
PULPROG	zg30
TD	32564
SOLVENT	CDCl3
NS	16
DS	2
SWH	8223.685 Hz
FIDRES	0.252539 Hz
AQ	1.9799412 sec
RG	90.5
DW	60.800 usec
DE	6.50 usec
TE	296.0 K
D1	2.00000000 sec
TD0	1

======== CHANNEL f1 ========

NUC1	1H
P1	9.40 usec
PL1	-4.00 dB
PL1W	19.93825150 W
SFO1	400.1324710 MHz
SI	32768
SF	400.1300000 MHz
WDW	EM
SSB	0
LB	0.30 Hz
GB	0
PC	1.00

Figure S 5

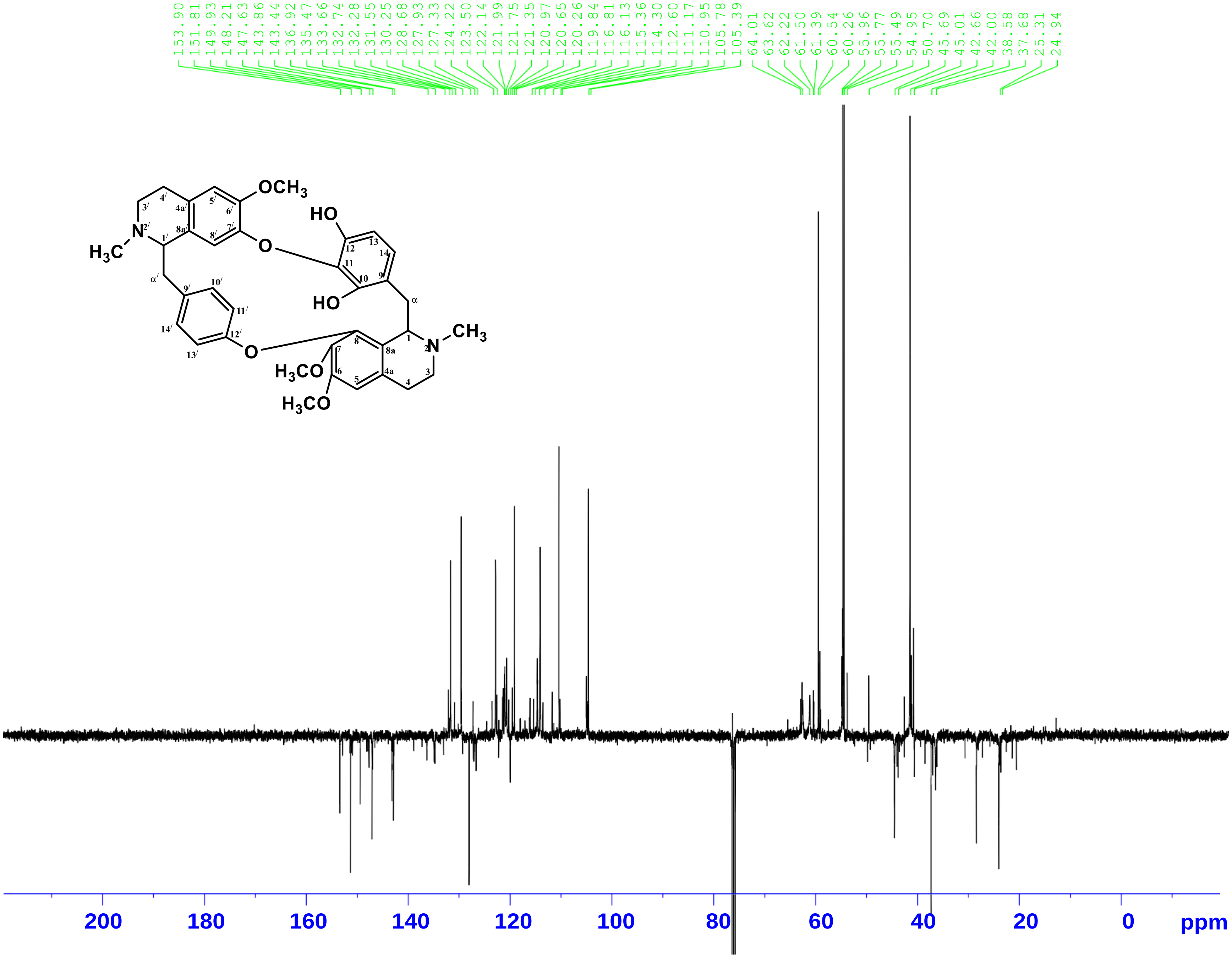

NAME D162831
EXPNO 2
PROCNO 1
Date_ 20120609
Time 4.53
INSTRUM spect
PROBHD 5 mm QNP 1H/13
PULPROG deptqgpsp.2
TD 32768
SOLVENT CDCl3
NS 3072
DS 4
SWH 24038.461 Hz
FIDRES 0.733596 Hz
AQ 0.6816244 sec
RG 2050
DW 20.800 usec
DE 6.50 usec
TE 295.6 K
CNST2 145.0000000
CNST12 1.5000000
D1 2.00000000 sec
D2 0.00344828 sec
D12 0.00002000 sec
D16 0.00020000 sec
D28 0.50000000 sec
TD0 1

======== CHANNEL f1 ========
NUC1 13C
P1 7.00 usec
P12 2000.00 usec
PL0 120.00 dB
PL1 -2.60 dB
PL0W 0.00000000 W
PL1W 61.34123230 W
SFO1 100.6228298 MHz
SP2 8.66 dB
SPNAM2 Crp60comp.4
SPOAL2 0.500
SPOFFS2 0.00 Hz

======== CHANNEL f2 ========
CPDPRG2 waltz16
NUC2 1H
P0 15.00 usec
P3 10.00 usec
P4 20.00 usec
PCPD2 80.00 usec
PL2 -4.00 dB
PL12 14.06 dB
PL13 120.00 dB
PL2W 19.93825150 W
PL12W 0.31166428 W
PL13W 0.00000000 W
SFO2 400.1316005 MHz

====== GRADIENT CHANNEL =====
GPNAM1 SINE.100
GPNAM2 SINE.100
GPNAM3 SINE.100
GPZ1 31.00 %
GPZ2 31.00 %
GPZ3 31.00 %
P16 1000.00 usec
SI 32768
SF 100.6127690 MHz
WDW EM
SSB 0
LB 1.00 Hz
GB 0
PC 1.40

Figure S 6

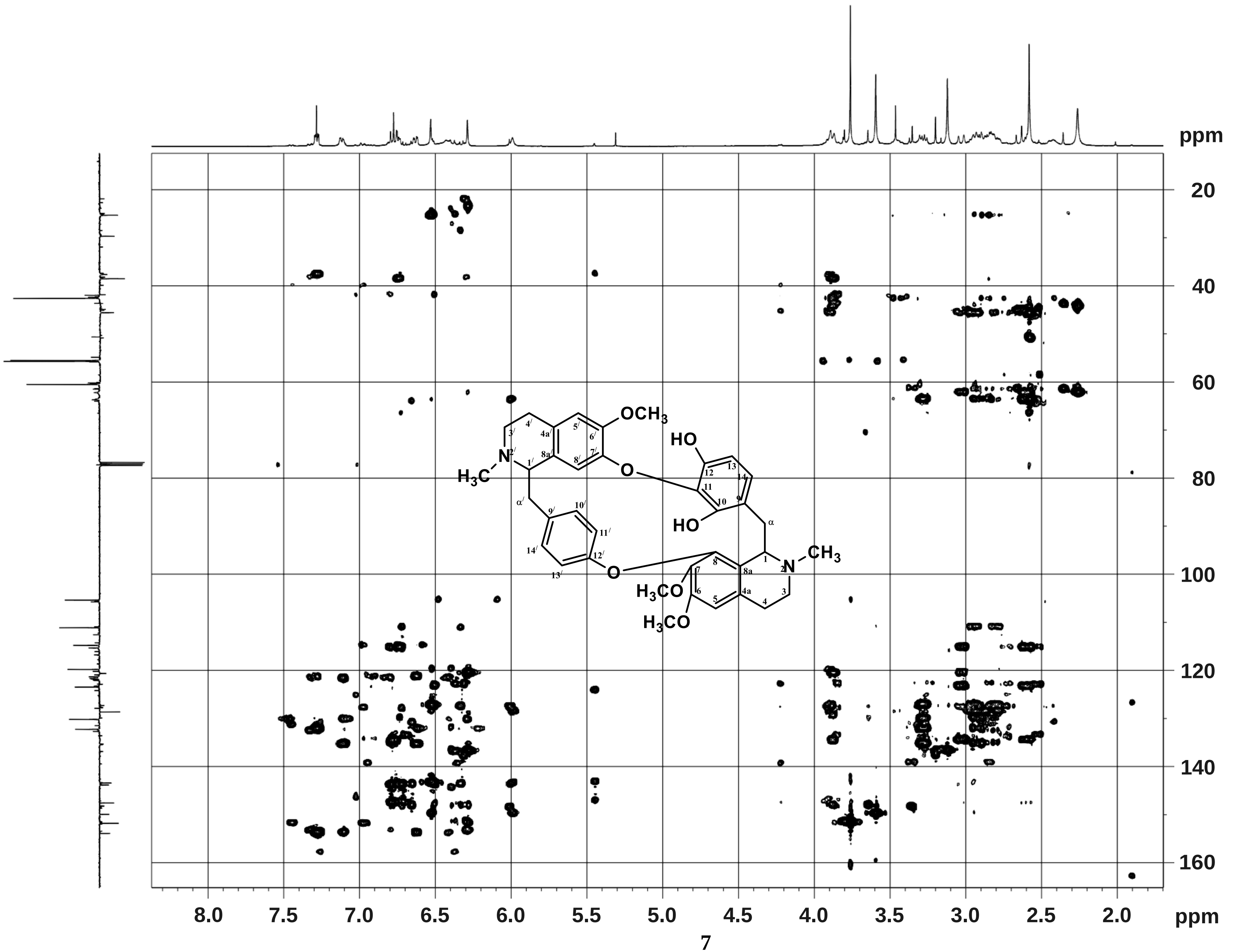


```
NAME              D162831
EXPNO                   3
PROCNO                  1
Date_            20120609
Time                18.36
INSTRUM             spect
PROBHD   5 mm QNP 1H/13
PULPROG      hmbcetgpl3nd
TD                   4096
SOLVENT             CDCl3
NS                     64
DS                     16
SWH              8012.820 Hz
FIDRES           1.956255 Hz
AQ              0.2556404 sec
RG                   2050
DW                 62.400 use
DE                   6.50 use
TE                  295.9 K
CNST6         120.0000000
CNST7         170.0000000
CNST13         10.0000000
CNST30          0.5981157
D0            0.00000300 sec
D1            1.50000000 sec
D6            0.05000000 sec
D16           0.00020000 sec
IN0           0.00002235 sec

======== CHANNEL f1 =======
NUC1                   1H
P1                   9.40 use
P2                  18.80 use
PL1                 -4.00 dB
PL1W          19.93825150 W
SFO1          400.1324008 MHz

======== CHANNEL f2 =======
NUC2                  13C
P3                   7.00 use
P24               2000.00 use
PL2                 -3.00 dB
PL2W          67.25932312 W
SFO2          100.6228138 MHz
SP7                  8.26 dB
SPNAM7       Crp60comp.4
SPOAL7              0.500
SPOFFS7              0.00 Hz

====== GRADIENT CHANNEL ===
GPNAM1         SMSQ10.100
GPNAM3         SMSQ10.100
GPNAM4         SMSQ10.100
GPNAM5         SMSQ10.100
GPNAM6         SMSQ10.100
GPZ1                80.00 %
GPZ3                14.00 %
GPZ4                -8.00 %
```

Figure S 7

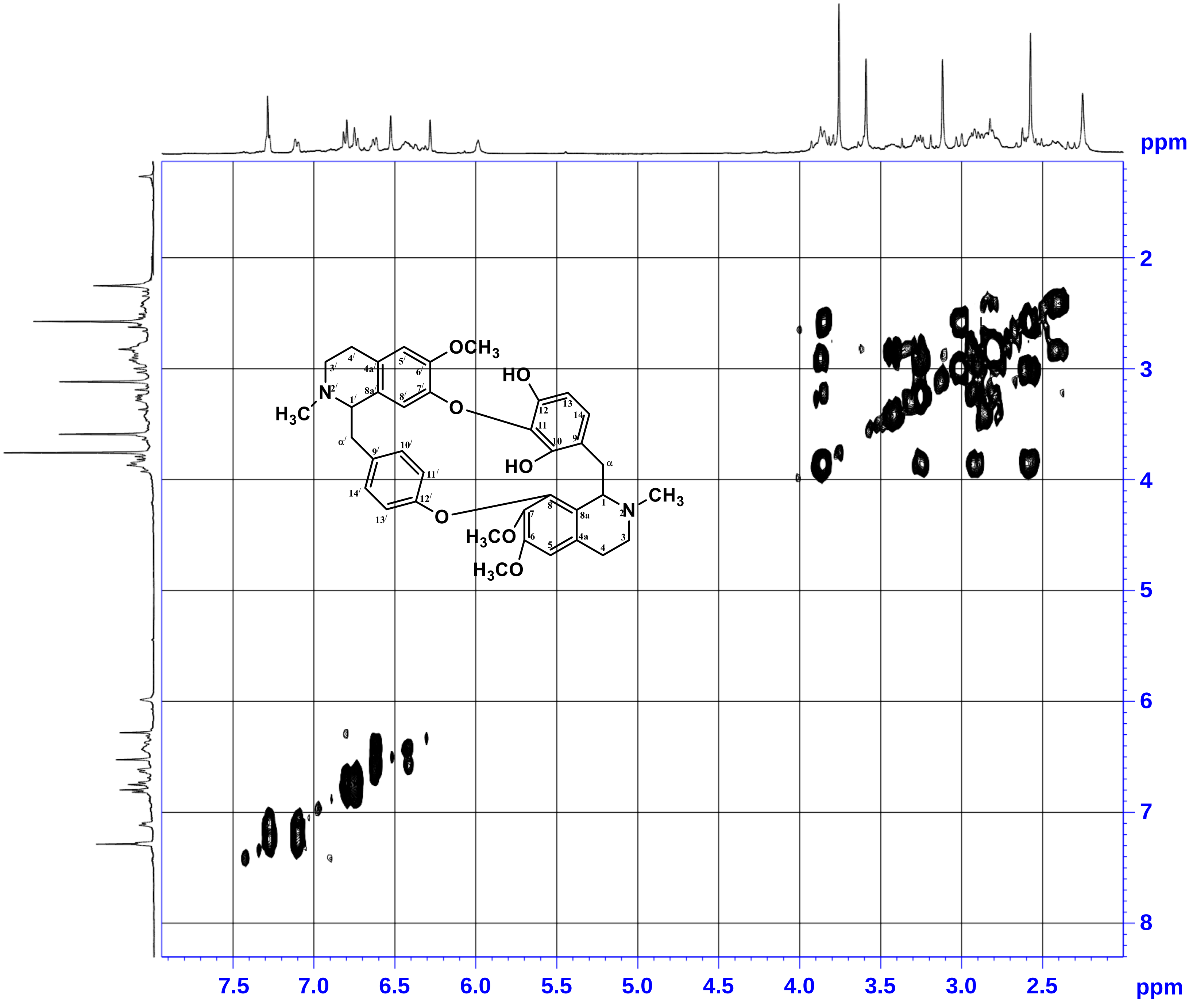

NAME	D166091
EXPNO	2
PROCNO	1
Date_	20120818
Time	5.28
INSTRUM	spect
PROBHD	5 mm QNP 1H/13
PULPROG	cosycggpmfqf.ptg
TD	2048
SOLVENT	CDCl3
NS	8
DS	8
SWH	8012.820 Hz
FIDRES	3.912510 Hz
AQ	0.1278452 sec
RG	2050
DW	62.400 usec
DE	6.50 usec
TE	295.6 K
D0	0.00000300 sec
D1	1.00000000 sec
D13	0.00000400 sec
D16	0.00020000 sec
IN0	0.00012495 sec
======== CHANNEL f1 ========	
NUC1	1H
P1	9.40 usec
PL1	-4.00 dB
PL1W	19.93825150 W
SFO1	400.1324008 MHz
====== GRADIENT CHANNEL =====	
GPNAM1	SMSQ10.100
GPNAM2	SMSQ10.100
GPNAM3	SMSQ10.100
GPNAM4	SMSQ10.100
GPNAM5	SMSQ10.100
GPZ1	16.00 %
GPZ2	12.00 %
GPZ3	40.00 %
GPZ4	27.13 %
GPZ5	23.17 %
P16	1000.00 usec
ND0	1
TD	128
SFO1	400.1324 MHz
FIDRES	62.520687 Hz
SW	20.000 ppm
FnMODE	QF
SI	1024
SF	400.1300000 MHz
WDW	SINE
SSB	0
LB	0.00 Hz
GB	0
PC	1.40
SI	1024
MC2	QF
SF	400.1300000 MHz
WDW	SINE
SSB	0
LB	0.00 Hz
GB	0

Figure S 8

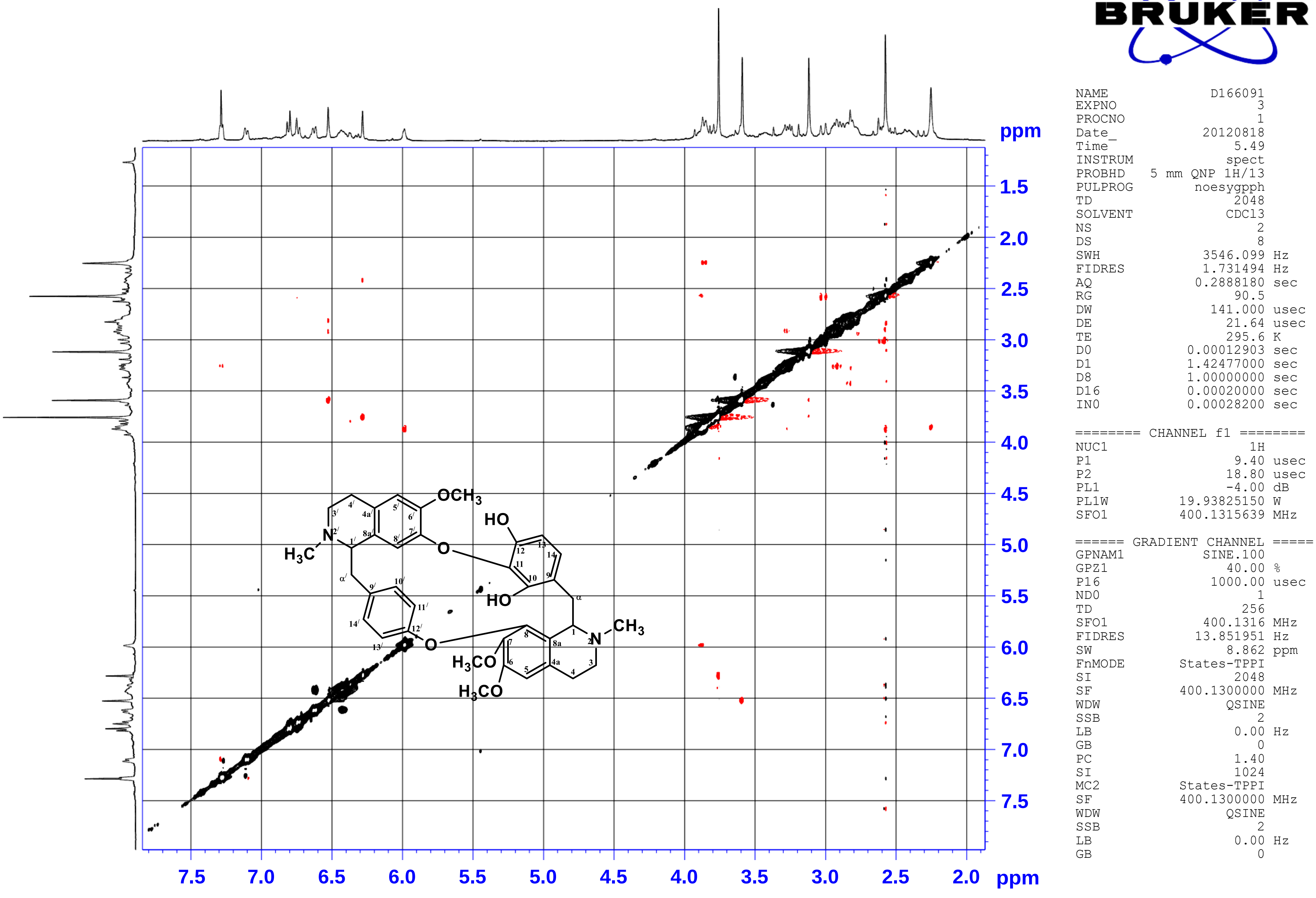

ppm
1.5
2.0
2.5
3.0
3.5
4.0
4.5
5.0
5.5
6.0
6.5
7.0
7.5
7.5
7.0
6.5
6.0
5.5
5.0
4.5
4.0
3.5
3.0
2.5
2.0
ppm
OCH3
HO
HO
H3C
N
O
O
CH3
N
H3CO
H3CO
NAME D166091
EXPNO 3
PROCNO 1
Date_ 20120818
Time 5.49
INSTRUM spect
PROBHD 5 mm QNP 1H/13
PULPROG noesygpph
TD 2048
SOLVENT CDCl3
NS 2
DS 8
SWH 3546.099 Hz
FIDRES 1.731494 Hz
AQ 0.2888180 sec
RG 90.5
DW 141.000 usec
DE 21.64 usec
TE 295.6 K
D0 0.00012903 sec
D1 1.42477000 sec
D8 1.00000000 sec
D16 0.00020000 sec
IN0 0.00028200 sec
======== CHANNEL f1 ========
NUC1 1H
P1 9.40 usec
P2 18.80 usec
PL1 -4.00 dB
PL1W 19.93825150 W
SFO1 400.1315639 MHz
====== GRADIENT CHANNEL =====
GPNAM1 SINE.100
GPZ1 40.00 %
P16 1000.00 usec
ND0 1
TD 256
SFO1 400.1316 MHz
FIDRES 13.851951 Hz
SW 8.862 ppm
FnMODE States-TPPI
SI 2048
SF 400.1300000 MHz
WDW QSINE
SSB 2
LB 0.00 Hz
GB 0
PC 1.40
SI 1024
MC2 States-TPPI
SF 400.1300000 MHz
WDW QSINE
SSB 2
LB 0.00 Hz
GB 0

Figure S9

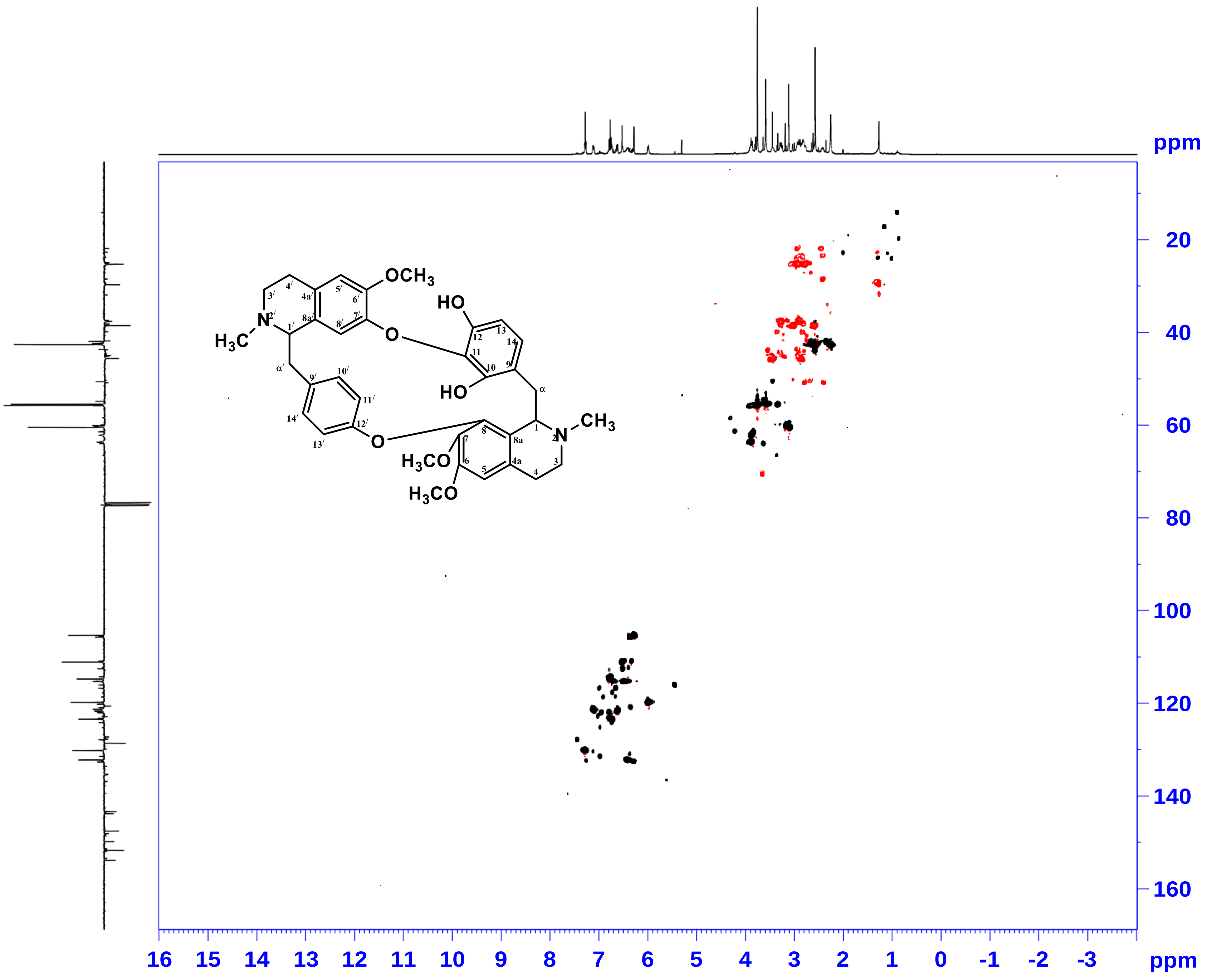

NAME	D162831	
EXPNO	4	
PROCNO	1	
Date_	20120608	
Time	13.18	
INSTRUM	spect	
PROBHD	5 mm QNP 1H/13	
PULPROG	hsqcedetgpsp.3	
TD	1024	
SOLVENT	CDCl3	
NS	8	
DS	16	
SWH	8012.820	Hz
FIDRES	7.825020	Hz
AQ	0.0639476	sec
RG	2050	
DW	62.400	usec
DE	6.50	usec
TE	296.0	K
CNST2	145.0000000	
D0	0.00000300	sec
D1	1.00000000	sec
D4	0.00172414	sec
D11	0.03000000	sec
D16	0.00020000	sec
D21	0.00360000	sec
IN0	0.00003000	sec
======== CHANNEL f1 ========		
NUC1	1H	
P1	9.40	usec
P2	18.80	usec
P28	0.00	usec
PL1	-4.00	dB
PL1W	19.93825150	W
SFO1	400.1324008	MHz
======== CHANNEL f2 ========		
CPDPRG2	garp4	
NUC2	13C	
P3	7.00	usec
P14	500.00	usec
P31	1900.00	usec
PCPD2	70.00	usec
PL0	120.00	dB
PL2	-3.00	dB
PL12	15.00	dB
PL0W	0.00000000	W
PL2W	67.25932312	W
PL12W	1.06598854	W
SFO2	100.6214217	MHz
SP3	8.26	dB
SP18	14.46	dB
SPNAM3	Crp60,0.5,20.1	
SPNAM18	Crp60_xfilt.2	
SPOAL3	0.500	
SPOAL18	0.500	
SPOFFS3	0.00	Hz

Table S2. MolDock docking energies (E_{dock}, kJ/mol) of chondrofolinol with inflammation-relevant protein targets.

protein structure	PDB code	E_{dock}	protein structure	PDB code	E_{dock}
Oa COX-1	3N8Z	+760.9	Hs MD-2	2E59	-90.3
Mm COX-2	6COX	+635.9	Hs MMP-1	1CGL	-119.3
Mm EPHX2	1CR6	-76.6	Hs MPO	4C1M	-106.7
Hs EPHX2	4HAI	-65.8	Mm NF-κB	3DO7	-74.9
Hs ERAP2	4JBS	-132.7	Hs p38MAPK	4FA2	-77.2
Hs GSTO1	5V3Q	-71.6	Hs PDE4B	3W5E	-98.6
Hs IKKβ	3BRV	-59.1	Hs PDE4D	5K32	-84.1
Xl IKKβ	3RZF	-103.0	Hs PI3Kγ	2A5U	-91.0
Hs IRAK4	5T1T	-100.3	Hs PLA2	1J1A	-92.9
Mm iNOS	1M8D	-102.5	Ss PLA2	2B03	-42.2
Hs JNK	4Y5H	-83.3	Hs PPAR-γ	3ADV	-91.0
Hs 5-LOX	3V99	-99.3			

Table S3. MolDock docking energies (E_{dock}, kJ/mol) of chondrofolinol with *Leishmania* protein targets.

Leishmania protein target	PDB code	E_{dock}	*Leishmania* protein target	PDB code	E_{dock}
Ldon CatB	A	-84.3	Lmaj NH	1EZR	-96.9
Lmaj CatB	A	-85.3	Lmaj OPB	2XE4	-107.9
Ldon Cyp	2HAQ	-87.4	Lmaj PDE1	2R8Q	-92.4
Lmaj dUTPase	2YAY	-83.8	Lmex PGI	1Q50	-89.2
Ldon DHODH	3C61	-70.2	Lmex PMM	2I55	-86.4
Lmaj DHODH	3MJY	-88.5	Lmaj PTR1	3H4V	-67.1
Lmex GAPDH	1A7K	-84.6	Lmex PYK	1PKL	-107.8
Lmex GPDH	1N1G	-94.3	Linf CYP51	3L4D	-99.5
Linf GLO2	2P18	-88.5	Linf TDR1	4AGS	-70.4
Lmaj MetRS	3KFL	-81.1	Lmex TIM	2Y61	-23.5
Ldon NMT	2WUU	-97.2	Linf TR	2YAU	-96.3
Lmaj NMT	2WSA	-122.0	Lmaj TyrRS	3P0J	-115.9
Linf PnC1	3K2J	-58.5	Lmaj UGPase	2OEF	-114.9
Lmaj NDKb	3NGU	-61.8			

[a] Prepared by structural homology to *Trypanosoma brucei* cathepsin B, PDB 3HHI (Ogungbe et al., 2014).
